# Functional connectivity characteristics of epilepsy with anxiety: A resting-state functional near-infrared spectroscopy study

**DOI:** 10.1097/MD.0000000000042660

**Published:** 2025-05-30

**Authors:** Xinxin Chen, Jiuhong You, Jinmei Li, Hui Ma, Mei Zhou, Cheng Huang

**Affiliations:** a Department of Rehabilitation Medicine, West China Hospital, Sichuan University, Chengdu, Sichuan Province, China; b Department of Rehabilitation Medicine, The First Affiliated Hospital, Sun Yat-sen University, Guangzhou, China; c Key Laboratory of Rehabilitation Medicine in Sichuan Province, West China Hospital, Sichuan University, Chengdu, Sichuan Province, China; d Department of Psychological Medicine, Center for Healthy Brain Ageing, Institute of Pychiatry, Pychology, and Neuroscience, King’s College London, London, United Kingdom; e Department of Neurology, West China Hospital, Sichuan University, Chengdu, Sichuan Province, China.

**Keywords:** anxiety, epilepsy, fNIRS, resting-state functional connectivity

## Abstract

Functional near-infrared spectroscopy (fNIRS) was applied to investigate the resting-state functional connectivity (RSFC) characteristics of the cerebral cortex in epileptic patients with and without anxiety. A total of 38 epileptic patients were recruited and divided into 2 groups according to the Generalized Anxiety Disorder 7-item score: epilepsy with anxiety (anxiety group) and epilepsy without anxiety (non-anxiety group). Resting-state fNIRS for 15 minutes was performed on each patient. Preprocessing of fNIRS data and RSFC analysis were performed in NirSpark software (Danyang Huichuang Medical Equipment Co., Ltd., China). Brain network was divided into 6 regions of interest (ROI). Based on the concentration of oxygenated hemoglobin in the time series, the RSFC strength was calculated. The RSFC between the 2 groups was compared in the bilateral prefrontal cortex, sensory cortex, and motor cortex. Epileptic patients with anxiety showed a decrease in group-averaged RSFC strength and ROI–ROI connectivity strength. The mean strength of RSFC and its standard deviations were 0.34 ± 0.14 for the anxiety group and 0.38 ± 0.15 for the non-anxiety group (*P* > .05, t = 0.854). The RSFC between part of the frontal and parietal channels in the anxiety group was significantly lower than that in the non-anxiety group (*P* < .05, t = 2.897). However, no significant difference was found after false discovery rate (FDR) correction. Before FDR correction, there was no significant difference except for the functional connectivity between the right prefrontal cortex and left motor cortex (*P* = .046, t = 2.064). However, there was no significant difference after FDR correction. fNIRS is an available imaging instrument for examining RSFC in various contexts. In this specific investigation, no significant difference was found in functional connectivity between epileptic patients with or without anxiety using resting-state fNIRS. Future studies should consider employing larger sample sizes or utilizing task-state fNIRS methodologies. By continuing to explore the capabilities of fNIRS in understanding brain connectivity and its association with anxiety in epilepsy patients, we can provide information for more effective diagnostic and therapeutic approaches, ultimately enhancing the care and management of these individuals.

**Key points**

- We firstly used functional near-infrared spectroscopy to study resting-state functional connectivity in epilepsy with and without anxiety.
- No significant difference was found in functional connectivity between epileptic patients with or without anxiety.
- Larger sample sizes or task-state functional near-infrared spectroscopy methodologies are needed in the future.

## 1. Introduction

Epilepsy is a group of cerebral diseases characterized by the production of epileptic seizures.^[1]^ Seizures present as transient signs and/or symptoms caused by abnormally hyperactive or synchronized neuronal activity in the brain.^[1]^ Seizures affect 10% of people worldwide and cause epilepsy in 1% to 2% of the global population.^[2]^ Epilepsy has adverse effects on a person’s social, occupational, physical and psychological functioning.^[3]^ Epileptic patients are 2 to 3 times more likely to have psychiatric comorbidities than the general public.^[4]^ Anxiety disorder ranks second among epilepsy psychiatric comorbidities following depression, affecting approximately 10% to 25% of epileptic patients.^[5]^ The clinical manifestations of epilepsy patients with anxiety are emotional control disorders, intellectual dysfunction, and behavioral disorders, accompanied by insomnia, tension, and anxious mood.^[6]^ However, depressive and psychotic disorders have received the most attention in studies on psychiatric comorbidities in epilepsy, while anxiety disorders and symptoms have often been ignored.^[7]^ Anxiety disorders were often described as a forgotten psychiatric comorbidity.^[4]^ A meta-analysis highlighted the considerable lack of studies on anxiety in epilepsy, an underappreciated comorbidity that can have serious consequences for quality of life and even mortality.^[8]^ Despite their significant impact on medical and psychosocial outcomes, anxiety disorders remain undiagnosed in routine clinical practice in up to two-thirds of people with epilepsy.^[9]^ Routine screening for anxiety in patients with epilepsy was included by the American Academy of Neurology as a quality indicator of treatment.^[10]^ The generic 7-item Generalized Anxiety Disorder Scale (GAD-7) is recommended for anxiety screening in epilepsy.^[11]^ In addition, Epilepsy Anxiety Survey Instrument and a shorter screening tool (brief Epilepsy Anxiety Survey Instrument) were developed to assess anxiety in patients with epilepsy.^[12]^ However, these are scale screening instruments and no neuroimaging tool has been developed to further objectively screen patients with anxiety and non-anxiety epilepsy.

There is spontaneous and natural neural activity when the brain is resting. Functional connectivity (FC) is the correlation or statistical dependence of neuronal activity between different brain areas in the time series.^[13]^ Functional magnetic resonance imaging (fMRI) is the most commonly used method when studying the resting-state functional connectivity (RSFC) characteristics of brain networks in people with anxiety.^[14–17]^ By using fMRI, 2 studies^[18,19]^ have reported that abnormal FC in the resting-state network was related to anxiety and depressive symptoms in epilepsy. Garcia et al^[18]^ proposed that decreased RSFC between visuospatial/dorsal attention and salient attention network and between the default mode network (DMN) and left executive control network were correlated with symptoms of depression and anxiety in idiopathic generalized epilepsy. Furthermore, temporal lobe epileptic patients showed abnormal RSFC between the medial temporal and frontal regions with respect to symptoms of anxiety and depression.^[19]^ However, fMRI equipment is large and expensive, takes a long time to operate, has low temporal resolution, and is not friendly to claustrophobic patients. Functional near-infrared spectroscopy (fNIRS) is a noninvasive and optical neuroimaging technique that can monitor the changes in deoxygenated hemoglobin (HbR), oxygenated hemoglobin (HbO_2_), and cortical hemodynamics in the cerebral cortex in real time.^[20]^ As a novel functional imaging tool for studying FC in the brain, fNIRS has attracted considerable attention. Compared with fMRI, fNIRS has the advantages of low cost, strong anti-noise ability, easy operation, portability, and is not affected by moving artifacts and any intrusion.^[21]^ Although both fMRI and fNIRS measure brain activity through relatively slow blood flow dynamics, fNIRS has a faster time sampling rate, meaning that the hemodynamic response features are more accurate. A review has shown that fNIRS can explore functional integration between local or whole brain regions by measuring inter-regional temporal synchronization.^[22]^ It is effective and reliable, providing a promising imaging tool for cognitive science and clinical practice. Wu et al^[21]^ used fNIRS to evaluate brain regions in anxious depressed group and non-anxious depressed group, and found significant differences in activation patterns in the right dorsolateral prefrontal cortex and right frontopolar cortex, suggesting that fNIRS may be a potential tool to improve the diagnostic accuracy of anxious states. At present, no studies have used fNIRS to explore the FC of the cerebral cortex in epileptic patients with anxiety.

This study firstly used fNIRS to investigate the RSFC of the cerebral cortex in epileptic patients with and without anxiety. It has been shown that patients with hereditary generalized epilepsy who have symptoms of anxiety and depression may be more likely to experience disrupted FC.^[18]^ Low synchronization of the connections between the right hippocampus and the prefrontal cortex was associated with higher depression and anxiety scores.^[21]^ The HbO_2_ in frontotemporal lobe of patients with anxiety was significantly lower than that of healthy people.^[23]^ Therefore, we hypothesized that epileptic patients with anxiety would show a decline in RSFC of the cerebral cortex compared with non-anxiety patients. It is helpful to explore a new evaluation method of cerebral cortical FC in epilepsy with anxiety and provide help for clinical diagnosis and intervention.

## 2. Materials and methods

### 2.1. Study population

This is a cross-sectional study. Subjects were recruited from West China Hospital of Sichuan University, China. The inclusion criteria were as follows: (1) age between 10 and 60 years; (2) epilepsy diagnosis identified by a neurologist; and (3) agreed to participate and provided written informed consent. Patients who (1) were unable to cooperate with the examination; (2) abused alcohol or other drugs; and (3) had other neurological diseases were excluded. All subjects were evaluated using GAD-7. Patients with GAD-7 scores higher than 4 were enrolled in the anxiety group, while the rest were enrolled in the non-anxiety group.

### 2.2. Resting-state acquisition of fNIRS

**Figure 1.**
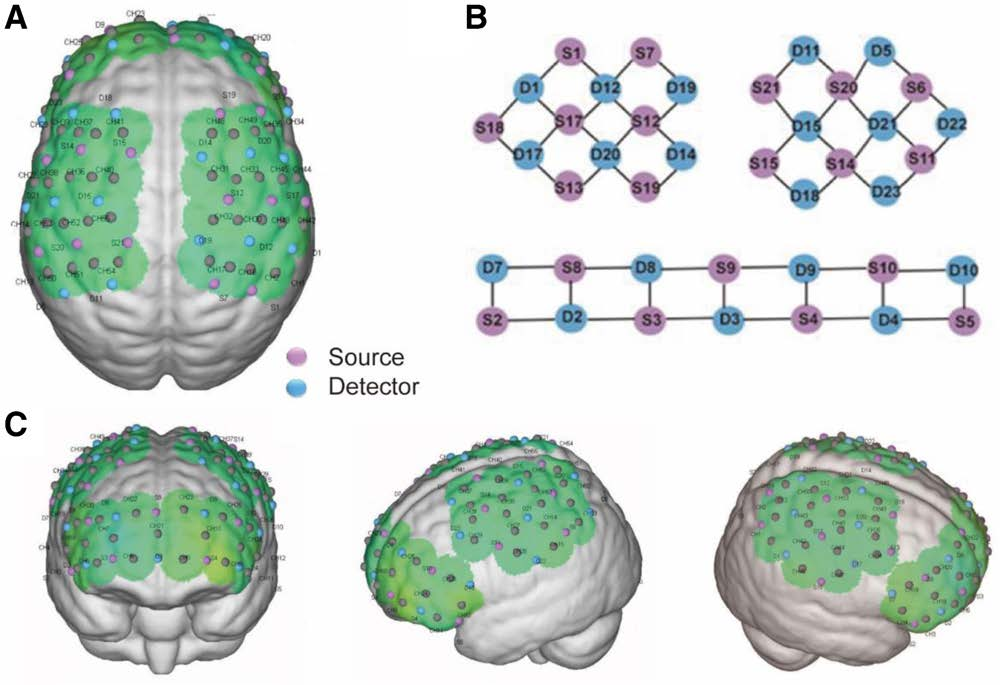
Placement of 21 light-emitting diodes (sources), 23 avalanche photodiodes (detectors), and 55 channels (CH). (A) An arrangement of optode that displays numbers on a three-dimensional model of the brain. (B) Two-dimensional distribution of the light source and detection probe. (C) Cerebral cortex where channels are covered.

The fNIRS signals of the subjects were obtained using a fNIRS system with multichannel (NirSmart-6000A, Danyang Huichuang Medical Equipment Co. Ltd, China). The acquisition cap was designed based on the 10/20 international system, which consists of 23 avalanche photodiodes (detectors) and 21 light-emitting diodes (sources), resulting in a total of 55 channels (CHs). The wavelengths of the light source probe were 730 nm and 850 nm. The whole CH sampling rate of the equipment was more than 11 Hz. Figure 1 shows the placement of the optode. The distance between the detection probe and light source probe was 30 mm, covering the bilateral prefrontal cortex (PFC), sensory cortex (SC), and motor cortex (MC) of the subjects’ cerebral cortex. The 55 CH were divided into 6 regions of interest (ROI) in the subject’s cerebral cortex (right SC, left SC, right MC, left MC, right PFC, left PFC). The correspondence between fNIRS acquisition CH and the Brodmann area is shown in Appendix A, Supplemental Digital Content, https://links.lww.com/MD/P80.

After entering the fNIRS assessment room, the subjects sat comfortably for 5 minutes to become familiar with the environment and eliminate their nervousness. The subjects were then given fNIRS head caps and instructed to close their eyes, empty their minds, and avoid physical activity. The subjects were reminded not to fall asleep during the resting-state fNIRS measurement. The evaluator closed the indoor lighting facilities and started to collect data for 15 minutes. For those who fell asleep, we had re-collected the resting-state fNIRS data.

### 2.3. Data analysis of fNIRS

#### 2.3.1. Preprocessing of fNIRS data

Preprocessing was performed in the Preprocess module of the NirSpark software package (Danyang Huichuang Medical Equipment Co., Ltd., China). First, motion artifacts were amended using the spline interpolation algorithm. Second, the physiological noise caused by cardiac activity, respiration, and Mayer waves was filtered using a 0.01 to 0.1 Hz bandpass filter. Finally, the filtered optical density data were converted into HbO_2_ concentration changes according to the modified Beer–Lambert law.

#### 2.3.2. Analysis of FC

The RSFC analysis was performed in the Network module of the NirSpark software (Danyang Huichuang Medical Equipment Co., Ltd., China). Oxygenated hemoglobin concentration time series were correlated between each CH pair using the Pearson correlation coefficient. Due to its normality characteristics, the z matrix was used for the next calculation step. By using Fisher r-to-z transformation method, we normalized the correlation coefficients to z values. Finally, the z values of the 55 × 55 FC matrix were averaged separately to determine the functional connections between and within networks.

### 2.4. Statistical analysis

SPSS software (version 26.0) was applied to test the normality of the baseline information of the subjects. The mean and standard deviation (SD) were used to express normally distributed data, and the independent sample *t* test was used for comparisons between groups. The median and interquartile range were used to express nonnormally distributed data, and the independent samples rank sum test was used for comparisons between groups. The percentage was used to express the counting data, and the chi-square test was used for comparisons between groups. The normality of the functional connection strength of each group was tested. NirSpark software was used to perform an independent sample *t* test for the mean RSFC strength of the ROI–ROI connection between the anxiety group and the non-anxiety group. The threshold of significance was *P* < .05. The false discovery rate (FDR) correction was performed.

## 3. Results

### 3.1. Characteristics of participants

A total of 38 epileptic patients who met the criteria were included. There were 18 patients in the anxiety group and 20 patients in the non-anxiety group. The baseline characteristics of the anxiety and non-anxiety groups are shown in Table 1. No significant difference was found in age, sex, duration of epilepsy, or type of epilepsy between the 2 groups.

**Table 1:** Baseline characteristics of the anxiety and non-anxiety groups.

	Anxiety group	Non-anxiety group	*P*-value
Age (years)	29.44 ± 9.414	27.10 ± 10.177	.467
Sex (male/female)	10/8	13/7	.552
GAD-7 scores (M, IQR)	8 (6)	0 (0)	.000
Duration of epilepsy (M, IQR) (years)	5 (11)	4 (3)	.391
Type of epilepsy (%)			.592
Focal onset epilepsy	72.2	60	
Generalized onset epilepsy	22.2	25	
Unknown onset epilepsy	5.6	15	

GAD-7 = Generalized Anxiety Disorder, IQR = interquartile range, M = median.

### 3.2. RSFC in epileptic patients with and without anxiety

**Figure 2.**
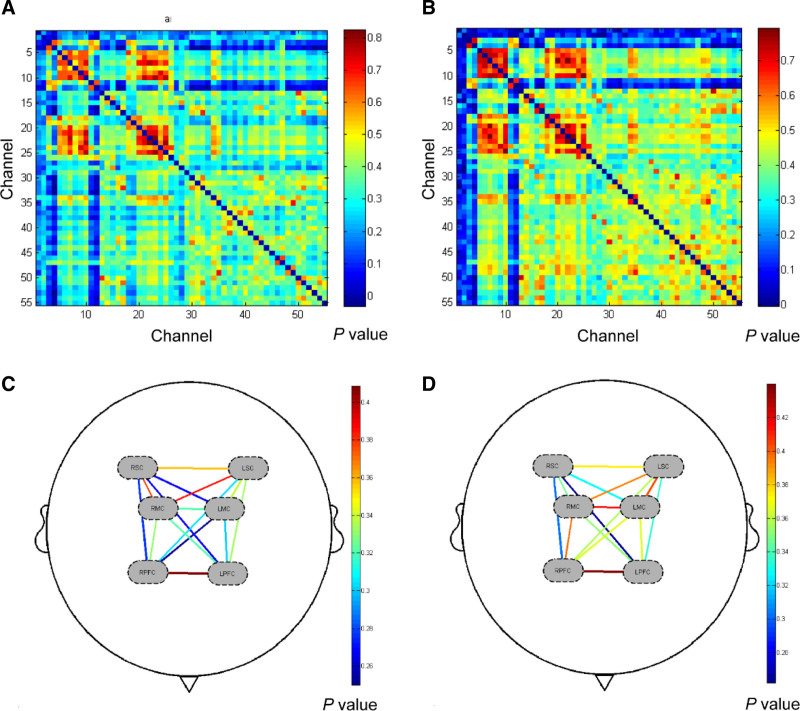
Spatial patterns of functional connectivity in the anxiety and non-anxiety groups. (A) Functional connectivity maps for the anxiety group. (B) Functional connectivity maps for the non-anxiety group. (C) The functional connectivity strength based on regions of interest division in the anxiety group. (D) The functional connectivity strength based on regions of interest division in the non-anxiety group. The number on the left of the color bar represents the mean HbO_2_, which means that the larger the mean HbO_2_ value of a channel, the closer the color is to red; otherwise, the closer it is to blue. The significance of functional connectivity strength in the 2 groups: *P* > .05.

**Figure 3.**
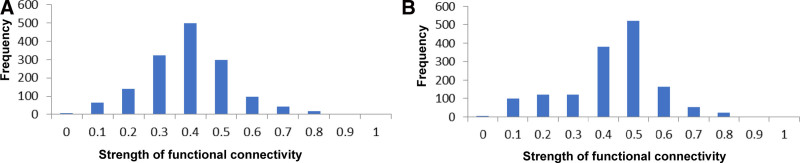
Histograms of the functional connectivity distribution. (A) The strength of functional connectivity in the anxiety group. (B) The strength of functional connectivity in the non-anxiety group. The unit of frequency is the number of times. The significance of functional connectivity strength in the 2 groups: *P* > .05.

**Figure 4.**
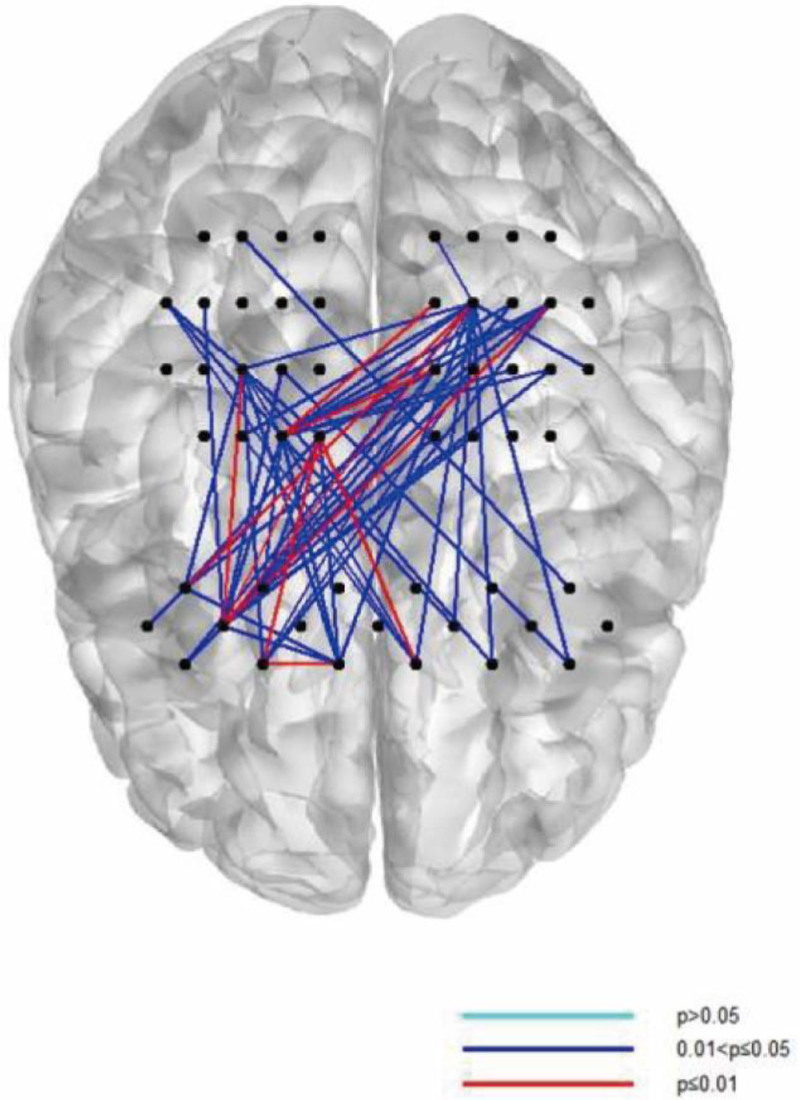
Differences in functional connectivity between the frontal and parietal channels in the 2 groups.

Figure 2A and B shows the group-averaged FC strength in the anxiety and non-anxiety groups. It was found that the averaged strength of brain FC in the anxiety group was lower than that in the non-anxiety group (Appendix B, Supplemental Digital Content, https://links.lww.com/MD/P81). However, no significant difference was found based on the connectivity of each CH in the 2 groups after FDR correction. Figure 2C and D shows the FC strength based on ROI division in the 2 groups. The ROI–ROI connectivity strength in the anxiety group was lower than that in the non-anxiety group. Figure 3 shows the histograms of the FC distribution. The functional connection strength of the anxiety group was mainly distributed in the range of 0.3 to 0.5, while that of the non-anxiety group was mainly distributed in the range of 0.4 to 0.6. Both groups showed a tendency of concentrated distribution. In quantity, the mean strength of RSFC and its SDs were 0.34 ± 0.14 for the anxiety group and 0.38 ± 0.15 for the non-anxiety group (*P* > .05, t = 0.854). The RSFC between part of the frontal and parietal CH in the anxiety group was significantly lower than that in the non-anxiety group (*P* < .05, t = 2.897) (Fig. 4). However, no significant difference was found after FDR correction.

Table 2 shows the significant differences in RSFC strength based on ROI–ROI connections in the 2 groups. It was found that the FC strength of ROI–ROI in the anxiety group was lower than that in the non-anxiety group, except for the FC between the left PFC and right SC and between the right MC and right SC. Before FDR correction, no significant difference was found except for the FC between the right PFC and left MC (Table 2). The anxiety group showed significantly lower connectivity between the right PFC and the left MC than the non-anxiety group in the ROI–ROI connection (*P* = .046, t = 2.064) (Table 2). However, there was no significant difference between the right PFC and left MC after FDR correction.

**Table 2:** Functional connectivity based on ROI–ROI connections in the 2 groups.

Region of interest	Anxiety group (mean ± SD)	Non-anxiety group (mean ± SD)	*P*	*P* (FDR corrected)	*t* test
LPFC–RPFC	0.41 ± 0.12	0.44 ± 0.15	.487	.880	0.703
LPFC–LMC	0.30 ± 0.15	0.36 ± 0.22	.288	.880	1.079
LPFC–RMC	0.32 ± 0.14	0.35 ± 0.21	.587	.880	0.549
LPFC–LSC	0.33 ± 0.14	0.34 ± 0.21	.958	.958	0.053
LPFC–RSC	0.27 ± 0.12	0.26 ± 0.16	.937	.958	-0.080
RPFC–LMC	0.25 ± 0.15	0.37 ± 0.19	.046	.694	2.064
RPFC–RMC	0.33 ± 0.16	0.40 ± 0.21	.299	.880	1.053
RPFC–LSC	0.30 ± 0.14	0.36 ± 0.20	.334	.880	0.979
RPFC–RSC	0.27 ± 0.13	0.30 ± 0.15	.576	.880	0.564
LMC–RMC	0.32 ± 0.23	0.41 ± 0.28	.283	.880	1.090
LMC–LSC	0.34 ± 0.22	0.40 ± 0.26	.428	.880	0.802
LMC–RSC	0.27 ± 0.20	0.33 ± 0.12	.414	.880	0.826
RMC–LSC	0.38 ± 0.21	0.40 ± 0.26	.910	.958	0.113
RMC–RSC	0.38 ± 0.22	0.35 ± 0.23	.680	.928	-0.415
LSC–RSC	0.36 ± 0.20	0.37 ± 0.22	.807	.958	0.246

FDR = false discovery rate, ROI = regions of interest, SD = standard deviation.

## 4. Discussion

In this research, fNIRS was applied to investigate the characteristics and differences in RSFC of the cerebral cortex in epileptic patients with and without anxiety. The results found that the overall FC strength of the anxiety group was lower than that in the non-anxiety group, and this trend was also detected in the FC strength based on the ROI–ROI connection. The mean strength of RSFC and its SDs were 0.34 ± 0.14 for the anxiety group and 0.38 ± 0.15 for the non-anxiety group (*P* > .05, t = 0.854). An examination of RSFC between certain frontal and parietal CH in the anxiety group did reveal a lower connectivity level compared to the non-anxiety group (*P* < .05, t = 2.897). However, this difference did not withstand correction for FDR. Before FDR correction, only the FC between the right PFC and left MC exhibited a significant difference (*P* = .046, t = 2.064). Nevertheless, this significance was not sustained after applying the FDR correction.

Functional connectivity is a basic measure of brain network changes in mental disorders,^[24]^ and reflects the temporal relationship of blood oxygen level-dependent signals in various regions of the brain.^[25]^ A brain network model suggested that anxiety and high trait anxiety were related to specific patterns of functional network impairment.^[26]^ Xu et al^[27]^ reported that low FC of the emotional network to the DMN and the executive control network and decoupling of the executive control network from the DMN could characterize anxiety. The connectivity patterns of the basolateral amygdala and centromedial amygdala were found to be significantly less distinct in adults with generalized anxiety disorder, indicating that their RSFC is disrupted.^[28]^ A study explored the association between cortical networks and state anxiety based on RSFC with fNIRS, suggesting that intrinsic cortical organization can predict state anxiety.^[29]^ These networks included the connections between the saliency network and frontoparietal network (FPN), FPN and the dorsal attention network, DMN and FPN, and DMN and saliency network, which were positively associated with state anxiety; connections between cortical regions of the DMN and dorsal attention network, as well as connections with the DMN, were negatively associated with state anxiety.^[29]^ Shi et al^[30]^ analyzed fMRI data and found that the functional coordination of bilateral inferior temporal gyrus in patients with temporal lobe epilepsy decreased, and the score of Hamilton anxiety scale was positively correlated with bilateral inferior temporal gyrus changes, indicating that FC changes occurred in temporal lobe epilepsy patients, and the changes were correlated with anxiety status. Therefore, it has been established that anxiety affects FC in cerebral cortical networks, and many studies tend to conclude that anxiety leads to a decrease in the strength of RSFC in cerebral cortical networks.^[31–33]^ In this study, we divided brain networks into ROI with reference to previous studies that selected brain regions that were more closely related to epilepsy and anxiety.^[34–37]^ Subdural electrode grids were placed in the primary somatosensory, parasylvian, and medial frontal cortex areas of the patient for surgical treatment of epilepsy.^[34]^ Patients with drug-resistant epilepsy both grasping of objects and the simply observing of those actions desynchronized subdural electrocorticographic alpha and beta rhythms as a marker of cortical activation in lateral premotor, primary somatosensory motor, and ventral prefrontal areas.^[35]^ Studies on anxiety have also found that anxiety was related to the low-frequency fluctuation amplitude of the somatosensory cortex and the somatic MC^[37]^ and was also closely related to the prefrontal cortex.^[36]^

Our study found that epileptic patients with anxiety showed a decrease in group-averaged RSFC strength. Anxiety weakens the synergy between brain regions, which may explain the cognitive decline and behavioral impairment in epileptic patients with anxiety. However, no significant difference was found in the FC strength between the anxiety and non-anxiety groups. The result was similar to that of a previous study.^[38]^ Wen et al^[38]^ explored the task and nontask brain activation differences for the assessment of depression and anxiety by fNIRS and indicated that the nontask may not be sufficient to separate psychiatric diseases from the healthy people. We also found that before FDR correction, the RSFC between part of the frontal and parietal CH in the anxiety group was significantly lower than that in the non-anxiety group. This may be because anxiety affects emotional control, behavior, and cognitive function in patients with epilepsy, which is consistent with previous findings that anxiety affects PFC flexibility and amygdala activity.^[39–41]^ PFC connectivity plays a role in behavioral and cognitive function.^[42]^ The PFC is thought to exert control by regulating activity in areas participating in threat detection, such as the amygdala, thus minimizing threat-induced anxiety and performance disruptions caused by target distraction.^[40]^ Yeung et al^[43]^ found that recent negative emotions were associated with decreased PFC function in healthy young people during working memory execution, suggesting that decreased PFC function was also present in non-clinical people with elevated levels of negative emotions. Clauss et al^[39]^ found connectivity between the PFC and limbic areas, and connections between different PFC areas were reduced in the anxiety group. The amygdala is the core region of the brain’s emotional circuits and extensively participates in emotional procedures, including memory, emotion perception, and regulation.^[44]^ However, anxiety dissociated the FC of the ventral and dorsal medial prefrontal cortex with the amygdala in the resting state.^[41]^ Amygdala activation is enhanced and PFC flexibility is decreased in anxious adults. In addition, before FDR correction, we found that the anxiety group showed significantly lower connectivity between the right PFC and the left MC than the non-anxiety group in the ROI–ROI connection. The PFC is an important area for higher cognitive processing, which provides top-down attention regulation, emotion, and cognitive control.^[45]^ The MC participates in voluntary campaigns for attention, execution and planning.^[46]^ There are 2 systems in the brain that control human emotions and motivation independently. The approach system mainly controls the behavioral motivation of reward, which involves the left frontal region.^[21]^ The avoidance system contains the right frontal region, which participates in modulating behavioral inhibition and is excessive in people with anxiety.^[21]^ Reduced connectivity between right PFC and left MC may affect the corresponding decline in function. After FDR correction, we were unable to uncover significant differences in the RSFC strength and the ROI–ROI connectivity strength between epileptic patients with or without anxiety using resting-state fNIRS. One fNIRS study^[47]^ showed that in older adults, symptoms of depression and anxiety were associated with decreased lateral PFC functioning when performing cognitive tasks. To gain deeper insights into the cortical activation and cerebral hemodynamics in epileptic patients with and without anxiety, further research is warranted. Future studies should consider employing larger sample sizes or exploring the potential benefits of utilizing task-state fNIRS methodologies. These advancements may offer a more comprehensive understanding of the distinct characteristics and functional patterns within epilepsy patients.

Our study has some limitations. First, as a cross-sectional study, the causal relationship cannot be determined. Second, because there is no consensus-based data-processing pipeline for effectively removing system noise and motion artifacts, fNIRS data may result in unreliable or difficult-to-interpret results. Third, fNIRS can only be used to observe changes in blood oxygen in the cortex and has a lower spatial resolution (2–3 cm). Thus, fNIRS is more limited for diseases that primarily cause changes in subcortical brain networks. Fourth, we did not use short separation CH in the preprocessing to isolate the hemodynamic response signal. Fifth, patients who previously taken epilepsy medications may bias the results. Sixth, neither HBO_2_ nor HbR had significant results in this study, we only used HBO_2_ as the fNIRS analysis indicator because of it has good stability and accuracy than HbR.^[48,49]^ Finally, as a pilot study, the total sample size in our study was not large enough due to participant’s availability and willingness. Nevertheless, most fNIRS studies that explore brain cortex functions have reported a small sample size (n = 6–12) and statistical analysis findings.^[50–54]^ The sample size might not affect our results.

In conclusion, fNIRS is an available imaging tool in RSFC studies. This study failed to identify FC differences between epileptic patients with or without anxiety by using resting-state fNIRS. More task-state fNIRS data are needed to better investigate the differences in cortical activation and cerebral hemodynamics in epileptic patients with and without anxiety. We will expand the sample size, include more network properties, use short separation CH, and apply task-state fNIRS data in the future. In this way, we can provide information for more effective diagnostic and therapeutic approaches, ultimately enhancing the care and management of people with epilepsy.

## Author contributions

**Data curation:** Jiuhong You.

**Formal analysis:** Xinxin Chen.

**Funding acquisition:** Cheng Huang.

**Methodology:** Xinxin Chen.

**Project administration:** Jiuhong You.

**Resources:** Jinmei Li.

**Software:** Xinxin Chen.

**Supervision:** Cheng Huang.

**Writing – original draft:** Xinxin Chen, Jiuhong You.

**Writing – review & editing:** Jinmei Li, Hui Ma, Mei Zhou, Cheng Huang.

## Supplementary Material


