# Supplementary material for: Functional connectivity characteristics of epilepsy with anxiety: A resting-state functional near-infrared spectroscopy study
Source: Medicine (Baltimore). 2025 May 30;104(22):e42660. doi: 10.1097/MD.0000000000042660 (PMC12425098; doi:10.1097/MD.0000000000042660)
Supplement: Supplementary file 1 [file medi-104-e42660-s001.pdf]

Table A. The correspondence between fNIRS acquisition channels and the Brodmann area.

Channel	Brodmann Area (Chris rorden' MRIcro)	ROI	Channel	Brodmann Area (Chris rorden' MRIcro)	ROI
1	40 - Supramarginal gyrus part of Wernicke's area	RSC	29	9 - Dorsolateral prefrontal cortex	LPFC
2	1 - Primary Somatosensory Cortex	RSC		44 - pars opercularis_ part of Broca's area	
	2 - Primary Somatosensory Cortex		30	4 - Primary Motor Cortex	RMC
	7 - Somatosensory Association Cortex			6 - Pre-Motor and Supplementary Motor Cortex	
	40 - Supramarginal gyrus part of Wernicke's area		31	6 - Pre-Motor and Supplementary Motor Cortex	RMC
3	45 - pars triangularis Broca's area	RPFC	32	4 - Primary Motor Cortex	RMC
	46 - Dorsolateral prefrontal cortex			6 - Pre-Motor and Supplementary Motor Cortex	
	47 - Inferior prefrontal gyrus		33	6 - Pre-Motor and Supplementary Motor Cortex	RMC
4	45 - pars triangularis Broca's area	RPFC	34	9 - Dorsolateral prefrontal cortex	RPFC
5	10 - Frontopolar area	RPFC		44 - pars opercularis_ part of Broca's area	
	11 - Orbitofrontal area		35	9 - Dorsolateral prefrontal cortex	RPFC
	46 - Dorsolateral prefrontal cortex		36	6 - Pre-Motor and Supplementary Motor Cortex	LPFC
6	10 - Frontopolar area	RPFC	37	8 - Includes Frontal eye fields	LPFC
	11 - Orbitofrontal area			9 - Dorsolateral prefrontal cortex	
7	10 - Frontopolar area	RPFC	38	4 - Primary Motor Cortex	LMC
8	10 - Frontopolar area	LPFC		6 - Pre-Motor and Supplementary Motor Cortex	
	11 - Orbitofrontal area		39	6 - Pre-Motor and Supplementary Motor Cortex	LPFC
9	10 - Frontopolar area	LPFC		9 - Dorsolateral prefrontal cortex	
	11 - Orbitofrontal area		40	6 - Pre-Motor and Supplementary Motor Cortex	LMC
	46 - Dorsolateral prefrontal cortex		41	6 - Pre-Motor and Supplementary Motor Cortex	LPFC
10	10 - Frontopolar area	LPFC		8 - Includes Frontal eye fields	
11	45 - pars triangularis Broca's area	LPFC	42	1 - Primary Somatosensory Cortex	RSC
	46 - Dorsolateral prefrontal cortex			3 - Primary Somatosensory Cortex	
	47 - Inferior prefrontal gyrus		43	3 - Primary Somatosensory Cortex	RMC

12	38 - Temporopolar area	LPFC
	45 - pars triangularis Broca's area	
13	40 - Supramarginal gyrus part of Wernicke's area	LSC
14	1 - Primary Somatosensory Cortex	LSC
	2 - Primary Somatosensory Cortex	
	3 - Primary Somatosensory Cortex	
	40 - Supramarginal gyrus part of Wernicke's area	
15	1 - Primary Somatosensory Cortex	LSC
	2 - Primary Somatosensory Cortex	
	40 - Supramarginal gyrus part of Wernicke's area	
16	1 - Primary Somatosensory Cortex	RSC
	7 - Somatosensory Association Cortex	
17	1 - Primary Somatosensory Cortex	RSC
	3 - Primary Somatosensory Cortex	
	4 - Primary Motor Cortex	
	5 - Somatosensory Association Cortex	
18	46 - Dorsolateral prefrontal cortex	RPFC
19	45 - pars triangularis Broca's area	RPFC
20	46 - Dorsolateral prefrontal cortex	RPFC
21	10 - Frontopolar area	RPFC
22	9 - Dorsolateral prefrontal cortex	RPFC
	10 - Frontopolar area	
23	9 - Dorsolateral prefrontal cortex	LPFC
	10 - Frontopolar area	
24	45 - pars triangularis Broca's area	LPFC
	46 - Dorsolateral prefrontal cortex	

	4 - Primary Motor Cortex	
44	4 - Primary Motor Cortex	RMC
	6 - Pre-Motor and Supplementary Motor Cortex	
45	4 - Primary Motor Cortex	RMC
	6 - Pre-Motor and Supplementary Motor Cortex	
46	1 - Primary Somatosensory Cortex	RSC
	2 - Primary Somatosensory Cortex	
	40 - Supramarginal gyrus part of Wernicke's area	
47	3 - Primary Somatosensory Cortex	RMC
	4 - Primary Motor Cortex	
	6 - Pre-Motor and Supplementary Motor Cortex	
	43 - Subcentral area	
48	6 - Pre-Motor and Supplementary Motor Cortex	RPFC
	8 - Includes Frontal eye fields	
49	8 - Includes Frontal eye fields	RPFC
	9 - Dorsolateral prefrontal cortex	
50	2 - Primary Somatosensory Cortex	LSC
	40 - Supramarginal gyrus part of Wernicke's area	
51	1 - Primary Somatosensory Cortex	LSC
	2 - Primary Somatosensory Cortex	
	3 - Primary Somatosensory Cortex	
	7 - Somatosensory Association Cortex	
52	3 - Primary Somatosensory Cortex	LMC
	4 - Primary Motor Cortex	
	6 - Pre-Motor and Supplementary Motor Cortex	
53	1 - Primary Somatosensory Cortex	LSC

25	46 - Dorsolateral prefrontal cortex	LPFC		3 - Primary Somatosensory Cortex	
26	45 - pars triangularis Broca's area	LPFC		4 - Primary Motor Cortex	
27	4 - Primary Motor Cortex	LMC	54	1 - Primary Somatosensory Cortex	LSC
	6 - Pre-Motor and Supplementary Motor Cortex			3 - Primary Somatosensory Cortex	
28	1 - Primary Somatosensory Cortex	LMC		5 - Somatosensory Association Cortex	
	3 - Primary Somatosensory Cortex			7 - Somatosensory Association Cortex	
	4 - Primary Motor Cortex		55	4 - Primary Motor Cortex	LMC
	43 - Subcentral area			6 - Pre-Motor and Supplementary Motor Cortex	

Abbreviations: PFC, prefrontal cortex; SC, sensory cortex; MC, motor cortex; R, right; L, left.
