# Supplementary material for: Functional connectivity characteristics of epilepsy with anxiety: A resting-state functional near-infrared spectroscopy study
Source: Medicine (Baltimore). 2025 May 30;104(22):e42660. doi: 10.1097/MD.0000000000042660 (PMC12425098; doi:10.1097/MD.0000000000042660)
Supplement: Supplementary file 2 [file medi-104-e42660-s002.pdf]

Supplementary appendix B. Functional connectivity based on channel-channel connections in the two groups.

Channel	non-anxiety Mean	anxiety Mean	non-anxiety Std	anxiety Std	p	p (FDR corrected)	T-test
1~2	0.185602	0.216194	0.796862	0.68181	0.900094	0.995741	-0.12643
1~3	0.054069	0.09645	0.139926	0.167157	0.400706	0.964667	-0.85043
1~4	0.028851	0.023926	0.087233	0.14777	0.902723	0.995741	0.123378
1~5	0.116789	0.176176	0.220067	0.229316	0.42084	0.964667	-0.81428
1~6	0.121359	0.190339	0.199554	0.288008	0.392538	0.964667	-0.86542
1~7	0.10157	0.235411	0.232038	0.279285	0.115539	0.924237	-1.61272
1~8	0.107475	0.137625	0.196203	0.277347	0.698888	0.98499	-0.38993
1~9	0.115604	0.172341	0.18475	0.243432	0.420859	0.964667	-0.81424
1~10	0.110137	0.20418	0.205273	0.281964	0.244228	0.924237	-1.18386
1~11	0.056648	0.128724	0.159438	0.199523	0.224462	0.924237	-1.236
1~12	0.027677	0.058781	0.139317	0.200043	0.578391	0.967027	-0.56083
1~13	0.008451	0.21612	0.338688	0.279523	0.047938	0.924237	-2.04771
1~14	0.209011	0.168898	0.306859	0.3138	0.692921	0.984023	0.398082
1~15	0.082737	0.155	0.211707	0.310621	0.403466	0.964667	-0.84541
1~16	0.303696	0.207914	0.428693	0.296569	0.425104	0.964667	0.807339
1~17	0.191971	0.178791	0.386663	0.323476	0.910464	0.995741	0.113247
1~18	0.066964	0.183693	0.157976	0.240356	0.082467	0.924237	-1.78635
1~19	0.062312	0.160096	0.183292	0.235667	0.15977	0.924237	-1.43553
1~20	0.079926	0.245052	0.21661	0.292306	0.054019	0.924237	-1.99181
1~21	0.060624	0.195236	0.191984	0.297174	0.102517	0.924237	-1.67542
1~22	0.099319	0.20231	0.222625	0.26829	0.204401	0.924237	-1.29255
1~23	0.102098	0.223276	0.2174	0.273251	0.137217	0.924237	-1.52012
1~24	0.069422	0.174667	0.163212	0.24976	0.129201	0.924237	-1.55287
1~25	0.124766	0.235712	0.258311	0.288407	0.218953	0.924237	-1.25114
1~26	0.051879	0.159452	0.160187	0.246187	0.11559	0.924237	-1.61249
1~27	0.180252	0.151189	0.324789	0.267448	0.766591	0.986311	0.29909
1~28	0.076321	0.128327	0.352696	0.246852	0.605618	0.967027	-0.52091
1~29	0.033316	0.111141	0.411234	0.366357	0.543647	0.967027	-0.61313
1~30	0.154735	0.221107	0.454213	0.30052	0.602947	0.967027	-0.52479
1~31	0.044626	0.165516	0.360455	0.303185	0.273537	0.924237	-1.11193
1~32	0.007057	0.176927	0.419509	0.30046	0.164118	0.924237	-1.42032
1~33	0.134858	0.178049	0.296685	0.325675	0.671302	0.975425	-0.42786
1~34	0.095466	0.313221	0.242205	0.34456	0.033286	0.924237	-2.23074
1~35	0.100705	0.10157	0.225615	0.498319	0.994658	0.998453	-0.00677
1~36	0.124691	0.162684	0.25381	0.333039	0.693054	0.984023	-0.3979
1~37	0.151271	0.128081	0.303654	0.276946	0.807857	0.993329	0.244988
1~38	0.159094	0.171461	0.237357	0.285519	0.884955	0.995741	-0.14572
1~39	0.050606	0.19287	0.342954	0.286353	0.176339	0.924237	-1.37921
1~40	0.153413	0.210481	0.248741	0.235423	0.473621	0.967027	-0.7242
1~41	0.088034	0.199701	0.265476	0.266344	0.204359	0.924237	-1.29267

1~42	0.104124	0.231688	0.329587	0.336527	0.245935	0.924237	-1.1795
1~43	0.15124	0.220804	0.480681	0.328553	0.609771	0.967027	-0.5149
1~44	0.117577	0.154251	0.282651	0.348451	0.722545	0.986311	-0.35785
1~45	0.051082	0.244196	0.219147	0.322284	0.041069	0.924237	-2.13629
1~46	0.050078	0.254197	0.374568	0.341744	0.089008	0.924237	-1.74789
1~47	0.077667	0.161488	0.246454	0.244172	0.300072	0.930327	-1.05142
1~48	0.162837	0.198549	0.302798	0.253482	0.69756	0.984674	-0.39174
1~49	0.093957	0.093123	0.236789	0.429828	0.994051	0.998453	0.007508
1~50	0.072647	0.286091	0.437304	0.378676	0.11839	0.924237	-1.59977
1~51	0.152996	0.243153	0.438913	0.332789	0.484001	0.967027	-0.70719
1~52	0.157946	0.211552	0.379812	0.251699	0.615488	0.967027	-0.50665
1~53	0.068277	0.143191	0.398978	0.359828	0.548838	0.967027	-0.60521
1~54	0.128087	0.222058	0.43791	0.311195	0.455431	0.964667	-0.75454
1~55	0.102896	0.175784	0.286432	0.310089	0.456203	0.964667	-0.75324
2~3	0.030746	0.062059	0.126373	0.19852	0.561455	0.967027	-0.58611
2~4	0.032558	0.012891	0.093267	0.207462	0.714799	0.986311	0.369944
2~5	0.073492	0.165771	0.202981	0.208627	0.17579	0.924237	-1.38101
2~6	0.074863	0.215173	0.197088	0.247685	0.060036	0.924237	-1.94166
2~7	0.122987	0.250853	0.260512	0.270262	0.146447	0.924237	-1.48425
2~8	0.065434	0.194699	0.189656	0.241004	0.073006	0.924237	-1.84682
2~9	0.055321	0.196423	0.175975	0.222077	0.035754	0.924237	-2.18154
2~10	0.111141	0.254078	0.205261	0.242833	0.057059	0.924237	-1.9659
2~11	0.03113	0.070413	0.11828	0.204704	0.481481	0.967027	-0.71394
2~12	0.02708	0.02655	0.10904	0.211632	0.992202	0.998253	0.009841
2~13	0.146682	0.240683	0.33114	0.278009	0.352554	0.962342	-0.94184
2~14	0.063022	0.261282	0.432949	0.279875	0.106574	0.924237	-1.65522
2~15	0.006571	0.150871	0.317407	0.277315	0.146321	0.924237	-1.48472
2~16	0.22869	0.280276	0.503695	0.467135	0.746171	0.986311	-0.32619
2~17	0.134735	0.362165	0.441855	0.395017	0.10456	0.924237	-1.66517
2~18	0.085756	0.153994	0.176234	0.230591	0.309418	0.936999	-1.031
2~19	0.07346	0.135923	0.200515	0.356625	0.504388	0.967027	-0.67436
2~20	0.1557	0.224199	0.264688	0.405514	0.537392	0.967027	-0.62272
2~21	0.071301	0.239515	0.17112	0.268837	0.030847	0.924237	-2.27249
2~22	0.150924	0.25131	0.264123	0.249194	0.237436	0.924237	-1.20141
2~23	0.140473	0.267528	0.223422	0.247945	0.105228	0.924237	-1.66185
2~24	0.070475	0.198942	0.15008	0.219718	0.040685	0.924237	-2.12315
2~25	0.16363	0.284083	0.261229	0.243936	0.151822	0.924237	-1.46418
2~26	0.076505	0.152858	0.135305	0.252398	0.263286	0.924237	-1.144
2~27	0.046601	0.17426	0.42437	0.301379	0.297123	0.928415	-1.05796
2~28	0.009688	0.101728	0.40726	0.242322	0.409547	0.964667	-0.83442
2~29	-0.00245	0.181647	0.400231	0.336886	0.136108	0.924237	-1.52456
2~30	0.097399	0.309629	0.485407	0.314342	0.122946	0.924237	-1.5796
2~31	0.195441	0.295501	0.347277	0.302926	0.352679	0.962342	-0.94159
2~32	0.110821	0.264748	0.417545	0.361493	0.234778	0.924237	-1.20838

2~33	0.028411	0.237774	0.32413	0.370503	0.071343	0.924237	-1.85814
2~34	0.116767	0.312476	0.242919	0.351777	0.051679	0.924237	-2.01264
2~35	0.114875	0.282885	0.2395	0.465112	0.180799	0.924237	-1.37703
2~36	0.196941	0.244089	0.242381	0.307258	0.600853	0.967027	-0.52783
2~37	0.157368	0.180379	0.321899	0.249094	0.808314	0.993329	-0.24439
2~38	0.11296	0.224154	0.358422	0.288174	0.302442	0.931798	-1.0462
2~39	0.078474	0.237278	0.356421	0.231919	0.116661	0.924237	-1.60759
2~40	0.078344	0.242253	0.321671	0.304143	0.116313	0.924237	-1.60918
2~41	0.088361	0.230733	0.298608	0.261094	0.128312	0.924237	-1.55661
2~42	0.105206	0.263626	0.326804	0.295332	0.127236	0.924237	-1.56116
2~43	0.197747	0.273347	0.46138	0.3778	0.586503	0.967027	-0.54884
2~44	0.101847	0.323193	0.251516	0.362737	0.038676	0.924237	-2.16291
2~45	0.095077	0.252609	0.25274	0.377519	0.14578	0.924237	-1.49444
2~46	0.055715	0.195911	0.357017	0.300025	0.201059	0.924237	-1.30238
2~47	0.090907	0.23633	0.249707	0.26675	0.091187	0.924237	-1.7356
2~48	0.11167	0.251243	0.366628	0.257012	0.187282	0.924237	-1.34424
2~49	0.136292	0.307583	0.250801	0.31515	0.070657	0.924237	-1.86287
2~50	0.188044	0.392689	0.431919	0.333443	0.113712	0.924237	-1.62115
2~51	0.092304	0.339064	0.484075	0.350604	0.083237	0.924237	-1.7817
2~52	0.182024	0.296452	0.468823	0.275685	0.372165	0.964667	-0.90369
2~53	0.160216	0.224651	0.452455	0.362793	0.633614	0.967027	-0.48073
2~54	0.271639	0.298022	0.382201	0.341489	0.824515	0.994197	-0.22336
2~55	0.172855	0.252405	0.267621	0.350132	0.433823	0.964667	-0.79152
3~4	0.708775	0.636671	0.165523	0.164541	0.187185	0.924237	1.344539
3~5	0.413298	0.55719	0.212169	0.184497	0.032871	0.924237	-2.2191
3~6	0.324448	0.343509	0.227231	0.239515	0.802727	0.993329	-0.25167
3~7	0.300914	0.397578	0.216928	0.267613	0.227235	0.924237	-1.22849
3~8	0.339132	0.326577	0.234608	0.225929	0.86782	0.995741	0.16762
3~9	0.378479	0.46893	0.236941	0.210553	0.223704	0.924237	-1.23807
3~10	0.325064	0.434289	0.205832	0.24329	0.142639	0.924237	-1.49882
3~11	0.549066	0.578517	0.250876	0.193358	0.690136	0.984023	-0.4019
3~12	0.485813	0.469034	0.239441	0.192814	0.814629	0.993329	0.236183
3~13	0.096346	0.147166	0.211805	0.143371	0.397599	0.964667	-0.85611
3~14	0.145473	0.127166	0.220132	0.183075	0.783426	0.993329	0.276919
3~15	0.23094	0.209466	0.215488	0.219883	0.76304	0.986311	0.303786
3~16	0.072297	0.100206	0.158262	0.160781	0.593393	0.967027	-0.53872
3~17	0.111776	0.093344	0.190212	0.151207	0.744706	0.986311	0.328141
3~18	0.444466	0.574014	0.248225	0.226569	0.102864	0.924237	-1.67367
3~19	0.456832	0.526897	0.283577	0.199968	0.389603	0.964667	-0.87086
3~20	0.269752	0.378592	0.235929	0.215087	0.147527	0.924237	-1.48017
3~21	0.256665	0.374772	0.250299	0.271434	0.171404	0.924237	-1.39553
3~22	0.251276	0.346639	0.244673	0.25448	0.246866	0.924237	-1.17714
3~23	0.240406	0.349814	0.239782	0.244976	0.173033	0.924237	-1.39011
3~24	0.418508	0.509263	0.268453	0.270423	0.306674	0.936999	-1.03695

3~25	0.262252	0.364034	0.213638	0.247774	0.182355	0.924237	-1.35978
3~26	0.375185	0.391608	0.276471	0.230742	0.844523	0.995741	-0.19753
3~27	0.203438	0.21359	0.208927	0.211693	0.882674	0.995741	-0.14863
3~28	0.264286	0.264394	0.296984	0.22413	0.999007	0.999007	-0.00125
3~29	0.235019	0.283538	0.18998	0.184495	0.43076	0.964667	-0.79685
3~30	0.137297	0.073586	0.207661	0.197248	0.340046	0.957459	0.966899
3~31	0.197485	0.147016	0.221588	0.217794	0.484293	0.967027	0.706714
3~32	0.145942	0.105331	0.199079	0.167174	0.502885	0.967027	0.676757
3~33	0.229339	0.094389	0.183071	0.208764	0.040673	0.924237	2.12328
3~34	0.25099	0.301528	0.239857	0.256529	0.534249	0.967027	-0.62757
3~35	0.237375	0.249832	0.228947	0.246401	0.87257	0.995741	-0.16154
3~36	0.241127	0.093235	0.173578	0.174632	0.012953	0.924237	2.614946
3~37	0.198659	0.206909	0.217635	0.191721	0.902489	0.995741	-0.12338
3~38	0.192763	0.134841	0.209862	0.144459	0.333661	0.957459	0.97993
3~39	0.240547	0.207331	0.183616	0.177958	0.575608	0.967027	0.564955
3~40	0.162005	0.102869	0.170225	0.157097	0.274868	0.924237	1.108802
3~41	0.217861	0.241152	0.214286	0.183816	0.722742	0.986311	-0.35759
3~42	0.159849	0.149145	0.214974	0.194193	0.873469	0.995741	0.160392
3~43	0.138603	0.09857	0.189161	0.207957	0.538183	0.967027	0.621506
3~44	0.209511	0.232339	0.241232	0.235644	0.770088	0.986311	-0.29447
3~45	0.248451	0.138544	0.197747	0.237753	0.12871	0.924237	1.554934
3~46	0.217273	0.233498	0.239809	0.234714	0.834589	0.994675	-0.21034
3~47	0.347401	0.377382	0.291956	0.270938	0.745587	0.986311	-0.32697
3~48	0.213236	0.120064	0.214114	0.20717	0.182284	0.924237	1.360008
3~49	0.23181	0.162858	0.231661	0.244409	0.377998	0.964667	0.892603
3~50	0.091944	0.110759	0.202592	0.165704	0.757434	0.986311	-0.31121
3~51	0.089658	0.142989	0.215661	0.182363	0.418642	0.964667	-0.81817
3~52	0.164862	0.090491	0.190738	0.162618	0.206685	0.924237	1.285907
3~53	0.100703	0.092974	0.256218	0.183716	0.916354	0.995741	0.105768
3~54	0.066002	0.096604	0.25191	0.146627	0.646545	0.972006	-0.46306
3~55	0.156523	0.094843	0.17913	0.194232	0.315277	0.942024	1.018414
4~5	0.292001	0.305687	0.255521	0.188656	0.851167	0.995741	-0.18903
4~6	0.218212	0.13842	0.256336	0.1768	0.276686	0.924237	1.104541
4~7	0.19732	0.175819	0.274803	0.232457	0.797241	0.993329	0.258829
4~8	0.233945	0.174594	0.259641	0.182096	0.42495	0.964667	0.807029
4~9	0.250632	0.265851	0.251838	0.180154	0.833262	0.994675	-0.21205
4~10	0.204627	0.206195	0.246118	0.16439	0.981914	0.998253	-0.02283
4~11	0.517844	0.531144	0.275845	0.163192	0.859508	0.995741	-0.17827
4~12	0.578927	0.582405	0.232902	0.219434	0.962596	0.998253	-0.04722
4~13	0.0122	0.114288	0.255563	0.115157	0.118224	0.924237	-1.61368
4~14	0.068004	0.063674	0.215934	0.18804	0.948079	0.998253	0.065575
4~15	0.181556	0.188224	0.238989	0.260659	0.934881	0.997713	-0.08228
4~16	0.014327	-0.00859	0.170047	0.220505	0.720344	0.986311	0.360819
4~17	0.046688	-0.0135	0.191547	0.175235	0.320817	0.945335	1.006659

4~18	0.30214	0.314176	0.27251	0.235389	0.885596	0.995741	-0.1449
4~19	0.437526	0.483644	0.257018	0.26591	0.590253	0.967027	-0.54333
4~20	0.166077	0.177629	0.2666	0.153597	0.869526	0.995741	-0.16563
4~21	0.137631	0.138573	0.270858	0.229316	0.990889	0.998253	-0.0115
4~22	0.143713	0.119481	0.272197	0.185684	0.753134	0.986311	0.316921
4~23	0.148892	0.138882	0.282976	0.202757	0.901957	0.995741	0.124061
4~24	0.313851	0.35827	0.279276	0.264475	0.618772	0.967027	-0.50193
4~25	0.16023	0.181223	0.230691	0.210777	0.772196	0.986311	-0.29169
4~26	0.344486	0.400682	0.303577	0.297072	0.5685	0.967027	-0.57555
4~27	0.106534	0.115394	0.210074	0.239116	0.903889	0.995741	-0.1216
4~28	0.267918	0.274667	0.30864	0.27866	0.944221	0.998253	-0.07046
4~29	0.111325	0.156189	0.226598	0.190568	0.515704	0.967027	-0.65646
4~30	0.071133	-0.01174	0.183653	0.238235	0.234989	0.924237	1.207826
4~31	0.09762	0.008601	0.223613	0.250487	0.254632	0.924237	1.157644
4~32	0.036433	-0.028	0.207724	0.214263	0.353183	0.962342	0.940592
4~33	0.105163	-0.01106	0.17376	0.231151	0.086379	0.924237	1.763057
4~34	0.190001	0.170191	0.288967	0.179582	0.799233	0.993329	0.256453
4~35	0.109833	0.12918	0.266972	0.237655	0.815649	0.993329	-0.23486
4~36	0.121158	0.048695	0.183968	0.200434	0.252827	0.924237	1.162137
4~37	0.110452	0.089709	0.221357	0.187022	0.758234	0.986311	0.310152
4~38	0.130724	0.036933	0.20957	0.190184	0.158861	0.924237	1.438745
4~39	0.15125	0.09908	0.237212	0.172686	0.447823	0.964667	0.767443
4~40	0.082555	0.071239	0.178639	0.183839	0.848578	0.995741	0.192313
4~41	0.10281	0.118033	0.245106	0.182701	0.830934	0.9943	-0.21506
4~42	0.069855	0.070782	0.230209	0.221172	0.989998	0.998253	-0.01262
4~43	0.064295	0.032824	0.188947	0.281192	0.691767	0.984023	0.4004
4~44	0.120345	0.145976	0.269889	0.280223	0.775709	0.988779	-0.28706
4~45	0.128413	0.084967	0.213822	0.235179	0.554532	0.967027	0.596564
4~46	0.150639	0.254608	0.213339	0.285016	0.208294	0.924237	-1.28126
4~47	0.349833	0.405257	0.288516	0.302925	0.567215	0.967027	-0.57747
4~48	0.107979	0.054803	0.250367	0.213479	0.488163	0.967027	0.700427
4~49	0.123154	0.040037	0.237441	0.229745	0.281201	0.924237	1.094046
4~50	0.005644	0.073352	0.240554	0.186205	0.342383	0.957459	-0.96217
4~51	0.02746	0.013231	0.219918	0.193355	0.834262	0.994675	0.210761
4~52	0.069144	0.044071	0.237781	0.218513	0.737951	0.986311	0.337164
4~53	0.024942	-0.03122	0.243535	0.192793	0.439304	0.964667	0.782041
4~54	0.025886	0.001975	0.233734	0.174159	0.725198	0.986311	0.354277
4~55	0.075349	0.001412	0.175814	0.194054	0.225774	0.924237	1.232438
5~6	0.735431	0.562518	0.151897	0.2414	0.011275	0.924237	2.671262
5~7	0.723897	0.666211	0.18858	0.246284	0.420191	0.964667	0.815425
5~8	0.679029	0.521386	0.14963	0.211396	0.011191	0.924237	2.674287
5~9	0.672302	0.645648	0.205656	0.140411	0.647377	0.972006	0.461274
5~10	0.674007	0.639353	0.19488	0.194102	0.586835	0.967027	0.548351
5~11	0.354202	0.478088	0.267292	0.211054	0.1244	0.924237	-1.57329

5~12	0.242734	0.280712	0.284493	0.233442	0.657698	0.972006	-0.4468
5~13	0.391688	0.339638	0.240323	0.161762	0.443958	0.964667	0.774046
5~14	0.435606	0.279925	0.240958	0.205059	0.039874	0.924237	2.1323
5~15	0.431825	0.323409	0.226523	0.215315	0.140309	0.924237	1.50789
5~16	0.276011	0.233857	0.283399	0.23868	0.625069	0.967027	0.49291
5~17	0.366539	0.237421	0.204394	0.24801	0.087221	0.924237	1.758157
5~18	0.707712	0.676158	0.173841	0.251447	0.652703	0.972006	0.453792
5~19	0.589941	0.522332	0.198704	0.228248	0.335474	0.957459	0.976213
5~20	0.651383	0.608866	0.217218	0.229075	0.560793	0.967027	0.587111
5~21	0.591582	0.611611	0.236739	0.218261	0.788582	0.993329	-0.27016
5~22	0.620342	0.546025	0.209415	0.233648	0.307963	0.936999	1.034152
5~23	0.566572	0.563513	0.236118	0.228454	0.967934	0.998253	0.040481
5~24	0.616357	0.583896	0.207774	0.187091	0.617392	0.967027	0.503913
5~25	0.605136	0.583879	0.210376	0.208162	0.756425	0.986311	0.312552
5~26	0.432727	0.418203	0.237113	0.249955	0.855228	0.995741	0.183766
5~27	0.389887	0.255502	0.287001	0.248979	0.133877	0.924237	1.533574
5~28	0.402123	0.295448	0.271281	0.255801	0.221792	0.924237	1.243306
5~29	0.422453	0.367063	0.244882	0.196967	0.450629	0.964667	0.762671
5~30	0.330097	0.290394	0.269582	0.197408	0.611132	0.967027	0.512932
5~31	0.358775	0.284796	0.290107	0.232823	0.39519	0.964667	0.860533
5~32	0.340914	0.272207	0.263365	0.168566	0.350699	0.960863	0.945516
5~33	0.364833	0.254913	0.257107	0.178848	0.132373	0.924237	1.541877
5~34	0.583585	0.514389	0.271467	0.227151	0.40271	0.964667	0.846784
5~35	0.496529	0.451788	0.199001	0.236951	0.531106	0.967027	0.632424
5~36	0.382735	0.222189	0.207482	0.177114	0.015149	0.924237	2.550637
5~37	0.366359	0.280364	0.318039	0.281899	0.385838	0.964667	0.877865
5~38	0.370288	0.197391	0.258446	0.18094	0.023643	0.924237	2.363128
5~39	0.4123	0.332179	0.27378	0.231965	0.339748	0.957459	0.967503
5~40	0.342575	0.210345	0.226863	0.209468	0.071081	0.924237	1.859937
5~41	0.411438	0.322095	0.256072	0.262758	0.295881	0.928415	1.060722
5~42	0.431689	0.347427	0.225127	0.173631	0.208354	0.924237	1.281089
5~43	0.341567	0.251935	0.309648	0.225047	0.318945	0.945335	1.010615
5~44	0.424604	0.392748	0.283993	0.211461	0.699903	0.98499	0.388543
5~45	0.423294	0.318985	0.243761	0.202976	0.16299	0.924237	1.424232
5~46	0.433216	0.348471	0.239461	0.214949	0.260604	0.924237	1.14295
5~47	0.46639	0.444714	0.22777	0.186866	0.751925	0.986311	0.318528
5~48	0.47672	0.269928	0.163933	0.242312	0.003657	0.924237	3.109095
5~49	0.472487	0.345156	0.210584	0.238074	0.088686	0.924237	1.74973
5~50	0.338927	0.291944	0.250718	0.183761	0.518243	0.967027	0.65247
5~51	0.312683	0.282272	0.26945	0.180706	0.68876	0.984023	0.403785
5~52	0.369273	0.206815	0.242067	0.189456	0.028289	0.924237	2.285301
5~53	0.327903	0.186661	0.320506	0.244312	0.138641	0.924237	1.514458
5~54	0.250941	0.257215	0.289992	0.178877	0.935853	0.997713	-0.08112
5~55	0.308372	0.202156	0.236937	0.188399	0.137779	0.924237	1.517879

6~7	0.74272	0.657845	0.165758	0.232698	0.200162	0.924237	1.305034
6~8	0.737283	0.61304	0.136277	0.271558	0.092199	0.924237	1.75261
6~9	0.636412	0.640096	0.213729	0.238624	0.960231	0.998253	-0.05021
6~10	0.718893	0.648981	0.15985	0.205136	0.246448	0.924237	1.178197
6~11	0.339798	0.33138	0.244826	0.241431	0.915748	0.995741	0.106537
6~12	0.194372	0.133704	0.269802	0.190467	0.433382	0.964667	0.79229
6~13	0.432966	0.351053	0.245978	0.207908	0.277781	0.924237	1.101987
6~14	0.454517	0.319792	0.256556	0.255581	0.114128	0.924237	1.619223
6~15	0.417161	0.333531	0.26208	0.26296	0.333325	0.957459	0.98062
6~16	0.301649	0.320066	0.273914	0.273289	0.837034	0.99525	-0.20718
6~17	0.404353	0.315571	0.215665	0.271987	0.269941	0.924237	1.120452
6~18	0.585104	0.449393	0.205001	0.301591	0.119265	0.924237	1.60445
6~19	0.503089	0.381695	0.204804	0.261492	0.117987	0.924237	1.601587
6~20	0.625291	0.540335	0.189314	0.265287	0.259735	0.924237	1.145073
6~21	0.70344	0.686667	0.225475	0.198561	0.810021	0.993329	0.242172
6~22	0.667193	0.587756	0.19286	0.23384	0.259012	0.924237	1.146843
6~23	0.607553	0.589749	0.249317	0.2213	0.818075	0.993329	0.23171
6~24	0.58016	0.541968	0.199238	0.254601	0.607839	0.967027	0.517694
6~25	0.62001	0.54647	0.197943	0.218789	0.283834	0.924237	1.087982
6~26	0.450263	0.390403	0.2217	0.274754	0.462655	0.964948	0.742411
6~27	0.382032	0.272782	0.2916	0.278719	0.246746	0.924237	1.177439
6~28	0.35742	0.273206	0.280642	0.257944	0.343731	0.957459	0.959452
6~29	0.456531	0.370794	0.234774	0.232586	0.26637	0.924237	1.128988
6~30	0.355469	0.337219	0.270226	0.236592	0.826824	0.994197	0.220375
6~31	0.378259	0.318981	0.296537	0.222971	0.494513	0.967027	0.69017
6~32	0.352708	0.334667	0.27793	0.212115	0.824809	0.994197	0.222984
6~33	0.365873	0.264633	0.29129	0.223617	0.241252	0.924237	1.191503
6~34	0.571988	0.51478	0.218885	0.25187	0.458623	0.964667	0.749169
6~35	0.530524	0.383094	0.183707	0.285212	0.06363	0.924237	1.913741
6~36	0.396125	0.265064	0.228571	0.205716	0.072565	0.924237	1.849799
6~37	0.412958	0.324508	0.321287	0.242	0.348478	0.957459	0.949939
6~38	0.392247	0.283116	0.284983	0.222291	0.199995	0.924237	1.305527
6~39	0.446042	0.364678	0.261064	0.210451	0.300712	0.930327	1.050009
6~40	0.393907	0.269672	0.226306	0.208399	0.087973	0.924237	1.753818
6~41	0.442761	0.336112	0.272613	0.195702	0.178798	0.924237	1.371207
6~42	0.428232	0.386176	0.256101	0.195849	0.576442	0.967027	0.563717
6~43	0.376344	0.306643	0.295487	0.226927	0.424076	0.964667	0.808566
6~44	0.439776	0.396762	0.294285	0.265641	0.640521	0.971576	0.470946
6~45	0.432832	0.309157	0.257004	0.25015	0.142355	0.924237	1.49992
6~46	0.452081	0.302691	0.219207	0.272625	0.069626	0.924237	1.870047
6~47	0.46908	0.38414	0.203578	0.268756	0.276504	0.924237	1.104969
6~48	0.497168	0.278022	0.183857	0.277498	0.00637	0.924237	2.897186
6~49	0.503923	0.297959	0.213842	0.287044	0.016123	0.924237	2.524832
6~50	0.389634	0.362821	0.262324	0.236739	0.743786	0.986311	0.329368

6~51	0.35549	0.346764	0.277695	0.231794	0.917365	0.995741	0.104484
6~52	0.397192	0.262284	0.262841	0.224529	0.099396	0.924237	1.691427
6~53	0.330359	0.281652	0.344419	0.26784	0.632274	0.967027	0.482639
6~54	0.282059	0.320431	0.305911	0.244224	0.674012	0.978321	-0.42411
6~55	0.360043	0.269598	0.247672	0.266754	0.285662	0.924237	1.083792
7~8	0.638552	0.498117	0.186082	0.285748	0.078152	0.924237	1.813159
7~9	0.588394	0.624958	0.199069	0.144078	0.524854	0.967027	-0.64214
7~10	0.748331	0.742105	0.130528	0.11954	0.87946	0.995741	0.152736
7~11	0.250475	0.376397	0.2183	0.256914	0.111152	0.924237	-1.63316
7~12	0.145616	0.146932	0.265053	0.210742	0.986681	0.998253	-0.01681
7~13	0.466941	0.378365	0.278367	0.221088	0.288274	0.924237	1.077842
7~14	0.487925	0.371296	0.25089	0.200831	0.12512	0.924237	1.570187
7~15	0.410076	0.403523	0.260208	0.235945	0.935902	0.997713	0.080986
7~16	0.384142	0.342721	0.301739	0.291733	0.670354	0.975425	0.429175
7~17	0.485418	0.403376	0.221675	0.242962	0.283572	0.924237	1.088584
7~18	0.620616	0.542174	0.214912	0.350461	0.418775	0.964667	0.820822
7~19	0.49374	0.439685	0.252218	0.339973	0.578698	0.967027	0.56037
7~20	0.770626	0.715228	0.17956	0.256457	0.441843	0.964667	0.777673
7~21	0.669155	0.726618	0.258557	0.155539	0.418528	0.964667	-0.81837
7~22	0.794888	0.768526	0.15248	0.095038	0.532039	0.967027	0.63098
7~23	0.671643	0.723902	0.211118	0.127955	0.368964	0.964667	-0.90983
7~24	0.548918	0.61948	0.203558	0.208192	0.298218	0.928415	-1.05552
7~25	0.691127	0.684983	0.172075	0.153226	0.908539	0.995741	0.115693
7~26	0.419533	0.452289	0.227238	0.267181	0.685442	0.983461	-0.40834
7~27	0.386922	0.282382	0.295144	0.294101	0.28208	0.924237	1.092017
7~28	0.351828	0.308697	0.278603	0.257882	0.62467	0.967027	0.49348
7~29	0.452425	0.414665	0.242074	0.265173	0.64904	0.972006	0.458935
7~30	0.426866	0.42365	0.286797	0.215693	0.969334	0.998253	0.038712
7~31	0.430865	0.355899	0.309687	0.272719	0.435845	0.964667	0.788017
7~32	0.416577	0.3844	0.306317	0.223322	0.716223	0.986311	0.366384
7~33	0.421127	0.314013	0.278713	0.26301	0.232382	0.924237	1.214715
7~34	0.597363	0.558199	0.211344	0.230971	0.588518	0.967027	0.545876
7~35	0.559127	0.515684	0.211672	0.288986	0.597737	0.967027	0.532375
7~36	0.433247	0.329089	0.229654	0.202212	0.148502	0.924237	1.476507
7~37	0.417856	0.372153	0.31883	0.303067	0.654258	0.972006	0.451612
7~38	0.403831	0.258576	0.299153	0.257225	0.119242	0.924237	1.595952
7~39	0.461142	0.406761	0.252757	0.245342	0.506222	0.967027	0.671447
7~40	0.442395	0.29905	0.220263	0.211435	0.048602	0.924237	2.041313
7~41	0.481863	0.408078	0.261424	0.267216	0.395655	0.964667	0.859678
7~42	0.487459	0.474219	0.272808	0.197819	0.866293	0.995741	0.169576
7~43	0.433573	0.37256	0.316542	0.220696	0.492033	0.967027	0.694591
7~44	0.453968	0.429274	0.292024	0.288492	0.795	0.993329	0.261758
7~45	0.491864	0.383593	0.268726	0.276518	0.229189	0.924237	1.223232
7~46	0.481476	0.369469	0.214722	0.27589	0.168827	0.924237	1.404204

7~47	0.474444	0.410194	0.217075	0.276721	0.428672	0.964667	0.800501
7~48	0.561332	0.381597	0.166392	0.250956	0.01258	0.924237	2.626845
7~49	0.537453	0.40799	0.225038	0.314145	0.15834	0.924237	1.44612
7~50	0.422537	0.419542	0.294996	0.236685	0.972856	0.998253	0.034264
7~51	0.431253	0.406261	0.277761	0.222709	0.763078	0.986311	0.303736
7~52	0.483756	0.316263	0.239419	0.199965	0.025782	0.924237	2.325727
7~53	0.345841	0.299001	0.366386	0.295357	0.669242	0.975425	0.430717
7~54	0.345939	0.364845	0.332191	0.234894	0.84227	0.995741	-0.20043
7~55	0.403432	0.342212	0.263863	0.195754	0.426306	0.964667	0.804647
8~9	0.669271	0.611422	0.19071	0.246969	0.421717	0.964667	0.812727
8~10	0.736007	0.584661	0.14723	0.275058	0.047626	0.924237	2.081452
8~11	0.352172	0.30716	0.232079	0.252068	0.57011	0.967027	0.573144
8~12	0.225904	0.190494	0.258235	0.21488	0.650766	0.972006	0.45651
8~13	0.395422	0.349407	0.240313	0.19526	0.524152	0.967027	0.643229
8~14	0.452549	0.270995	0.24165	0.234696	0.02471	0.924237	2.344101
8~15	0.447938	0.313529	0.248461	0.238285	0.098227	0.924237	1.69753
8~16	0.280995	0.267563	0.268435	0.282677	0.881445	0.995741	0.1502
8~17	0.365313	0.293475	0.213905	0.252013	0.348305	0.957459	0.950283
8~18	0.575358	0.423795	0.209628	0.270348	0.059999	0.924237	1.941958
8~19	0.483886	0.393702	0.185454	0.270214	0.234186	0.924237	1.209944
8~20	0.595736	0.449718	0.183819	0.287197	0.067373	0.924237	1.886072
8~21	0.660614	0.582924	0.212837	0.259136	0.317375	0.942605	1.013945
8~22	0.642106	0.469037	0.177531	0.303334	0.043745	0.924237	2.116331
8~23	0.664695	0.528666	0.196279	0.266777	0.079806	0.924237	1.802739
8~24	0.586731	0.482675	0.194844	0.234556	0.144143	0.924237	1.493029
8~25	0.621691	0.508171	0.18925	0.222705	0.098062	0.924237	1.698393
8~26	0.469447	0.336605	0.191164	0.234105	0.0623	0.924237	1.923909
8~27	0.409445	0.23956	0.299622	0.269	0.07538	0.924237	1.831048
8~28	0.406753	0.285693	0.27929	0.246616	0.167274	0.924237	1.409479
8~29	0.456609	0.371995	0.263123	0.213374	0.286869	0.924237	1.081037
8~30	0.342602	0.284347	0.248338	0.223355	0.453991	0.964667	0.756975
8~31	0.351738	0.267347	0.325036	0.215506	0.348107	0.957459	0.951727
8~32	0.353072	0.267713	0.256505	0.201351	0.265128	0.924237	1.131976
8~33	0.393457	0.215404	0.265974	0.226413	0.033616	0.924237	2.209116
8~34	0.564523	0.435293	0.224164	0.257802	0.107035	0.924237	1.652959
8~35	0.533422	0.352934	0.182914	0.242758	0.013281	0.924237	2.604748
8~36	0.412762	0.222421	0.232664	0.180959	0.008335	0.924237	2.791908
8~37	0.38233	0.257052	0.333012	0.239379	0.195817	0.924237	1.318036
8~38	0.41184	0.226675	0.273461	0.211156	0.026327	0.924237	2.316643
8~39	0.449404	0.301006	0.271512	0.189859	0.057312	0.924237	1.967567
8~40	0.388693	0.230148	0.216987	0.18821	0.022039	0.924237	2.39323
8~41	0.411712	0.272095	0.309314	0.221277	0.12198	0.924237	1.583825
8~42	0.399089	0.313533	0.24226	0.215765	0.260049	0.924237	1.144304
8~43	0.357983	0.260886	0.278146	0.229177	0.251063	0.924237	1.166548

8~44	0.453222	0.378172	0.263376	0.215659	0.346212	0.957459	0.954469
8~45	0.433261	0.229485	0.268657	0.24634	0.020325	0.924237	2.427676
8~46	0.416654	0.320871	0.211054	0.236912	0.195741	0.924237	1.318266
8~47	0.459281	0.389837	0.196534	0.212644	0.302437	0.931798	1.046213
8~48	0.500787	0.221646	0.175399	0.230031	0.000153	0.226498	4.231626
8~49	0.481928	0.262645	0.230521	0.24607	0.007452	0.924237	2.835991
8~50	0.355965	0.328063	0.243021	0.25222	0.730521	0.986311	0.347122
8~51	0.348447	0.328934	0.25171	0.215278	0.799903	0.993329	0.255354
8~52	0.405465	0.216914	0.249814	0.193756	0.014166	0.924237	2.578307
8~53	0.32602	0.253721	0.337226	0.250913	0.462416	0.964948	0.74281
8~54	0.290631	0.30021	0.290014	0.252715	0.914611	0.995741	-0.10798
8~55	0.335217	0.248584	0.260934	0.232397	0.289187	0.924237	1.075771
9~10	0.707241	0.728198	0.199272	0.131921	0.707907	0.986311	-0.37765
9~11	0.490276	0.477287	0.217516	0.211853	0.853442	0.995741	0.18606
9~12	0.33362	0.272273	0.239423	0.203107	0.402759	0.964667	0.846695
9~13	0.303947	0.342564	0.267803	0.213789	0.628819	0.967027	-0.48756
9~14	0.409898	0.32896	0.260065	0.180132	0.27737	0.924237	1.102944
9~15	0.459931	0.36171	0.243119	0.202838	0.187473	0.924237	1.343641
9~16	0.22813	0.269751	0.261698	0.228486	0.606556	0.967027	-0.51955
9~17	0.262474	0.297393	0.218624	0.206107	0.616597	0.967027	-0.50506
9~18	0.579656	0.523807	0.251389	0.246053	0.494195	0.967027	0.690682
9~19	0.445698	0.453188	0.245256	0.233627	0.923952	0.997713	-0.09613
9~20	0.498279	0.531964	0.246268	0.222786	0.66235	0.973458	-0.4403
9~21	0.511911	0.625112	0.235014	0.138007	0.082855	0.924237	-1.784
9~22	0.524713	0.567632	0.220147	0.178099	0.516023	0.967027	-0.65596
9~23	0.516099	0.620378	0.251521	0.154173	0.13735	0.924237	-1.51959
9~24	0.732588	0.731227	0.146369	0.115372	0.974976	0.998253	0.031586
9~25	0.612519	0.636536	0.220178	0.138413	0.693546	0.984023	-0.39723
9~26	0.51322	0.513938	0.158781	0.206446	0.990421	0.998253	-0.01209
9~27	0.40264	0.286826	0.313124	0.255346	0.222709	0.924237	1.240788
9~28	0.415975	0.348057	0.284129	0.208863	0.411175	0.964667	0.831496
9~29	0.428454	0.408748	0.258501	0.244166	0.811032	0.993329	0.240857
9~30	0.302149	0.348015	0.257529	0.176971	0.530952	0.967027	-0.63266
9~31	0.291597	0.289587	0.299036	0.225244	0.981629	0.998253	0.023187
9~32	0.261641	0.301452	0.269347	0.143175	0.568532	0.967027	-0.57667
9~33	0.336546	0.232532	0.26725	0.199858	0.180391	0.924237	1.366927
9~34	0.468767	0.491081	0.240231	0.212715	0.764622	0.986311	-0.30169
9~35	0.430423	0.406721	0.190167	0.26098	0.749127	0.986311	0.322251
9~36	0.369161	0.263058	0.212261	0.2089	0.129864	0.924237	1.550103
9~37	0.38452	0.320625	0.298045	0.24638	0.478914	0.967027	0.715503
9~38	0.352189	0.229093	0.292157	0.225847	0.158235	0.924237	1.440969
9~39	0.405925	0.371335	0.305428	0.195994	0.677586	0.98051	0.41951
9~40	0.338616	0.277851	0.233454	0.186049	0.384378	0.964667	0.880595
9~41	0.377519	0.345087	0.283654	0.194889	0.681405	0.98051	0.414119

9~42	0.319161	0.342261	0.271354	0.153804	0.74603	0.986311	-0.32682
9~43	0.311076	0.28861	0.282609	0.162484	0.763137	0.986311	0.304041
9~44	0.368182	0.389129	0.302327	0.233476	0.811484	0.993329	-0.24031
9~45	0.378573	0.285412	0.263567	0.20028	0.225504	0.924237	1.233744
9~46	0.338322	0.299009	0.271878	0.221569	0.630476	0.967027	0.485197
9~47	0.405322	0.42122	0.229569	0.184368	0.816588	0.993329	-0.23364
9~48	0.39403	0.288627	0.189009	0.226376	0.126678	0.924237	1.563525
9~49	0.399695	0.305198	0.246768	0.246191	0.245752	0.924237	1.179966
9~50	0.25346	0.344239	0.253551	0.192035	0.22553	0.924237	-1.2331
9~51	0.267653	0.354865	0.269425	0.160199	0.229082	0.924237	-1.2266
9~52	0.34732	0.26722	0.260868	0.175111	0.270301	0.924237	1.120944
9~53	0.269582	0.272028	0.352758	0.22353	0.979586	0.998253	-0.02579
9~54	0.207368	0.30133	0.2854	0.192429	0.238509	0.924237	-1.20013
9~55	0.266391	0.267165	0.259792	0.177764	0.9916	0.998253	-0.0106
10~11	0.350881	0.400152	0.210596	0.286458	0.553828	0.967027	-0.59854
10~12	0.221894	0.197127	0.248222	0.217416	0.74667	0.986311	0.325522
10~13	0.448821	0.399728	0.23469	0.211663	0.504464	0.967027	0.674242
10~14	0.486606	0.383441	0.237839	0.198696	0.15798	0.924237	1.441878
10~15	0.454357	0.408942	0.245495	0.189409	0.530673	0.967027	0.633096
10~16	0.359389	0.333409	0.282646	0.28692	0.780393	0.993045	0.280903
10~17	0.42698	0.388423	0.212985	0.235533	0.599369	0.967027	0.529995
10~18	0.614147	0.557278	0.197594	0.316878	0.506418	0.967027	0.671136
10~19	0.501215	0.450708	0.202131	0.298753	0.541837	0.967027	0.615898
10~20	0.695242	0.620809	0.163608	0.262702	0.296221	0.928415	1.059964
10~21	0.692592	0.765344	0.205383	0.117158	0.184469	0.924237	-1.35762
10~22	0.706651	0.70039	0.156838	0.120385	0.891902	0.995741	0.136862
10~23	0.729419	0.773804	0.198188	0.074385	0.360683	0.964667	-0.93133
10~24	0.663197	0.678471	0.137959	0.158326	0.752473	0.986311	-0.3178
10~25	0.783826	0.757013	0.119793	0.133921	0.518825	0.967027	0.651557
10~26	0.539799	0.461174	0.187705	0.237396	0.262562	0.924237	1.138183
10~27	0.448235	0.32627	0.320365	0.296504	0.232802	0.924237	1.213601
10~28	0.402735	0.335516	0.286737	0.245819	0.44548	0.964667	0.771441
10~29	0.48679	0.441681	0.273289	0.257785	0.605003	0.967027	0.521803
10~30	0.404351	0.381703	0.278248	0.181954	0.770994	0.986311	0.293278
10~31	0.427263	0.352841	0.28364	0.263886	0.409492	0.964667	0.834519
10~32	0.400708	0.39588	0.262874	0.156638	0.946327	0.998253	0.067792
10~33	0.403868	0.290506	0.290663	0.265069	0.218933	0.924237	1.251198
10~34	0.5738	0.560781	0.209759	0.209644	0.849526	0.995741	0.191093
10~35	0.551364	0.478873	0.164487	0.293938	0.363877	0.964667	0.924157
10~36	0.448766	0.321236	0.248261	0.252383	0.125453	0.924237	1.56876
10~37	0.444238	0.416398	0.34836	0.318029	0.799208	0.993329	0.256261
10~38	0.445392	0.285976	0.298807	0.283138	0.100992	0.924237	1.683195
10~39	0.471411	0.44409	0.280319	0.276918	0.764612	0.986311	0.301706
10~40	0.458767	0.334724	0.22101	0.234727	0.102097	0.924237	1.677555

10~41	0.46771	0.440625	0.296754	0.273747	0.772444	0.986311	0.291366
10~42	0.462117	0.38504	0.268433	0.176081	0.308165	0.936999	1.033713
10~43	0.435129	0.336754	0.297323	0.190899	0.229166	0.924237	1.225419
10~44	0.457279	0.437986	0.296195	0.251212	0.830779	0.9943	0.21526
10~45	0.469235	0.361696	0.292192	0.238925	0.225411	0.924237	1.233422
10~46	0.468399	0.340236	0.254345	0.264468	0.136733	0.924237	1.522051
10~47	0.458142	0.44808	0.20775	0.212283	0.883531	0.995741	0.147537
10~48	0.51815	0.324002	0.165433	0.279345	0.015975	0.924237	2.570745
10~49	0.553231	0.350866	0.200685	0.28447	0.01773	0.924237	2.50821
10~50	0.42877	0.412483	0.234721	0.218547	0.826632	0.994197	0.220624
10~51	0.396149	0.414346	0.289445	0.170645	0.81281	0.993329	-0.23881
10~52	0.478182	0.317577	0.249194	0.222548	0.044134	0.924237	2.085916
10~53	0.39141	0.295762	0.323329	0.283239	0.340951	0.957459	0.965064
10~54	0.351525	0.372495	0.298991	0.191815	0.796628	0.993329	-0.25982
10~55	0.409678	0.328283	0.227966	0.204964	0.256777	0.924237	1.152339
11~12	0.657929	0.641774	0.235941	0.193245	0.819911	0.993933	0.229329
11~13	0.111612	0.211768	0.165004	0.179261	0.081297	0.924237	-1.7935
11~14	0.195957	0.121307	0.226496	0.183252	0.274828	0.924237	1.108896
11~15	0.325057	0.296717	0.222354	0.227772	0.700437	0.98499	0.387814
11~16	0.090542	0.036692	0.158065	0.217234	0.384668	0.964667	0.880052
11~17	0.080751	0.0763	0.15017	0.182173	0.934695	0.997713	0.082514
11~18	0.309059	0.400606	0.257149	0.294161	0.312803	0.937522	-1.02371
11~19	0.357281	0.389172	0.286673	0.27548	0.729291	0.986311	-0.34877
11~20	0.213644	0.298568	0.198979	0.254747	0.257171	0.924237	-1.15137
11~21	0.245865	0.307893	0.252494	0.285759	0.481981	0.967027	-0.71049
11~22	0.2234	0.329792	0.214313	0.2532	0.169326	0.924237	-1.40252
11~23	0.239777	0.320723	0.21805	0.277169	0.328106	0.957459	-0.99299
11~24	0.579388	0.54934	0.245288	0.224382	0.697019	0.984674	0.392478
11~25	0.317404	0.373577	0.224342	0.284054	0.500905	0.967027	-0.67992
11~26	0.52418	0.479298	0.233638	0.206103	0.535969	0.967027	0.624913
11~27	0.275319	0.111344	0.208126	0.190637	0.016203	0.924237	2.522784
11~28	0.363646	0.345054	0.366435	0.217304	0.848664	0.995741	0.192406
11~29	0.218121	0.204486	0.228009	0.244022	0.859675	0.995741	0.178058
11~30	0.10448	0.100037	0.187725	0.201267	0.944268	0.998253	0.070396
11~31	0.106166	0.095472	0.204888	0.197303	0.871055	0.995741	0.16348
11~32	0.066593	0.096493	0.183645	0.163812	0.601282	0.967027	-0.52721
11~33	0.161273	0.047948	0.187584	0.173093	0.06173	0.924237	1.92833
11~34	0.193342	0.279367	0.233562	0.22556	0.256867	0.924237	-1.15212
11~35	0.171671	0.193245	0.199868	0.208719	0.7468	0.986311	-0.32535
11~36	0.174803	0.096541	0.198715	0.180436	0.213713	0.924237	1.265807
11~37	0.18895	0.124174	0.220318	0.264005	0.415313	0.964667	0.824094
11~38	0.207663	0.045597	0.198106	0.153179	0.008216	0.924237	2.797585
11~39	0.204737	0.173015	0.219626	0.221007	0.660238	0.972672	0.443249
11~40	0.141788	0.133281	0.162014	0.187122	0.881443	0.995741	0.150203

11~41	0.176925	0.160557	0.224285	0.231253	0.826073	0.994197	0.221348
11~42	0.127846	0.177505	0.207739	0.228256	0.487067	0.967027	-0.70221
11~43	0.139788	0.10549	0.19355	0.21267	0.605877	0.967027	0.520537
11~44	0.178556	0.224676	0.24787	0.269362	0.585917	0.967027	-0.5497
11~45	0.20393	0.129737	0.218674	0.207712	0.292064	0.927465	1.069273
11~46	0.16759	0.170712	0.243389	0.274498	0.970556	0.998253	-0.03717
11~47	0.285822	0.310331	0.294231	0.247535	0.784008	0.993329	-0.27616
11~48	0.150958	0.080617	0.1658	0.16643	0.200687	0.924237	1.303476
11~49	0.166033	0.127651	0.179218	0.213582	0.550861	0.967027	0.602131
11~50	0.093111	0.158087	0.154872	0.181934	0.242205	0.924237	-1.18905
11~51	0.108197	0.140316	0.160399	0.193433	0.579437	0.967027	-0.55928
11~52	0.159406	0.107457	0.179778	0.17692	0.376148	0.964667	0.896108
11~53	0.088087	0.086238	0.221717	0.164636	0.977096	0.998253	0.02891
11~54	0.073244	0.086055	0.176534	0.190851	0.831005	0.9943	-0.21497
11~55	0.12537	0.086835	0.172747	0.14381	0.462588	0.964948	0.742524
12~13	0.028874	0.125041	0.17443	0.175899	0.099634	0.924237	-1.69019
12~14	0.108447	0.082467	0.260037	0.238688	0.751096	0.986311	0.319631
12~15	0.280583	0.283989	0.248345	0.292876	0.969277	0.998253	-0.03878
12~16	0.040404	-0.01688	0.18805	0.205621	0.375611	0.964667	0.897129
12~17	0.029603	0.01961	0.175959	0.141765	0.849314	0.995741	0.191367
12~18	0.232799	0.268442	0.29824	0.218004	0.679534	0.98051	-0.41648
12~19	0.316735	0.362009	0.297863	0.299653	0.643663	0.972006	-0.46651
12~20	0.122883	0.153015	0.25005	0.160894	0.665355	0.97441	-0.43612
12~21	0.102923	0.116666	0.292802	0.207763	0.86978	0.995741	-0.16511
12~22	0.11359	0.113417	0.26378	0.218949	0.998266	0.999007	0.002188
12~23	0.163042	0.135942	0.261483	0.214199	0.73055	0.986311	0.347083
12~24	0.383674	0.378421	0.290569	0.24917	0.952891	0.998253	0.05949
12~25	0.213329	0.184934	0.274337	0.253542	0.7432	0.986311	0.33015
12~26	0.516451	0.541933	0.248076	0.290727	0.772332	0.986311	-0.29151
12~27	0.179268	0.092685	0.250237	0.232813	0.278427	0.924237	1.100482
12~28	0.335101	0.39043	0.342036	0.282136	0.592288	0.967027	-0.54034
12~29	0.150794	0.153494	0.276274	0.193799	0.972673	0.998253	-0.0345
12~30	0.048711	0.002324	0.212843	0.215167	0.508805	0.967027	0.667349
12~31	0.074769	0.029647	0.208748	0.178081	0.480622	0.967027	0.712705
12~32	0.035543	0.009388	0.194342	0.171598	0.664265	0.973774	0.437636
12~33	0.110937	-0.00863	0.199099	0.181478	0.061897	0.924237	1.927027
12~34	0.162406	0.130331	0.25505	0.177163	0.658776	0.972006	0.445291
12~35	0.080708	0.102767	0.25004	0.178983	0.75873	0.986311	-0.30949
12~36	0.110406	0.049306	0.193314	0.170827	0.311071	0.936999	1.027433
12~37	0.099553	0.109877	0.250661	0.205849	0.891159	0.995741	-0.13781
12~38	0.099306	0.04333	0.228264	0.151185	0.384459	0.964667	0.880445
12~39	0.157999	0.102718	0.249287	0.179253	0.442308	0.964667	0.776875
12~40	0.096337	0.117884	0.161687	0.221392	0.732069	0.986311	-0.34504
12~41	0.110628	0.109529	0.263016	0.208565	0.988782	0.998253	0.014159

12~42	0.059911	0.081369	0.204404	0.225957	0.76031	0.986311	-0.3074
12~43	0.070509	0.025836	0.224284	0.232845	0.550881	0.967027	0.602101
12~44	0.104839	0.12001	0.266062	0.274135	0.863611	0.995741	-0.17301
12~45	0.100675	0.06472	0.2119	0.194368	0.590475	0.967027	0.543001
12~46	0.118077	0.162165	0.234082	0.264623	0.589087	0.967027	-0.54504
12~47	0.245931	0.311074	0.296208	0.276202	0.489172	0.967027	-0.69879
12~48	0.07969	0.023664	0.211015	0.176734	0.383776	0.964667	0.881723
12~49	0.099819	0.041718	0.238174	0.186883	0.412052	0.964667	0.829924
12~50	0.014338	0.063742	0.173705	0.172757	0.38594	0.964667	-0.87767
12~51	0.013841	0.06506	0.188973	0.209473	0.433236	0.964667	-0.79254
12~52	0.087016	0.081967	0.209302	0.210601	0.941399	0.998253	0.074027
12~53	0.031142	0.05627	0.212662	0.233089	0.730216	0.986311	-0.34753
12~54	0.038545	0.017434	0.171516	0.216882	0.739956	0.986311	0.334483
12~55	0.057596	0.035994	0.164498	0.164871	0.68878	0.984023	0.403757
13~14	0.464719	0.426805	0.429848	0.431062	0.787844	0.993329	0.271124
13~15	0.468727	0.425607	0.325754	0.41112	0.720858	0.986311	0.360124
13~16	0.373567	0.420631	0.506302	0.333446	0.740113	0.986311	-0.33427
13~17	0.494572	0.363961	0.4601	0.464361	0.390101	0.964667	0.869933
13~18	0.394505	0.281863	0.224763	0.205059	0.116687	0.924237	1.607473
13~19	0.346349	0.286313	0.279694	0.228232	0.476233	0.967027	0.719903
13~20	0.495407	0.427555	0.23107	0.228481	0.369603	0.964667	0.908603
13~21	0.439441	0.396984	0.300714	0.211284	0.615138	0.967027	0.507405
13~22	0.516892	0.440096	0.263605	0.211416	0.332	0.957459	0.983345
13~23	0.486756	0.477058	0.270117	0.208191	0.902859	0.995741	0.122914
13~24	0.349576	0.3836	0.247546	0.233333	0.66641	0.974995	-0.43465
13~25	0.487115	0.490482	0.274866	0.246455	0.968641	0.998253	-0.03959
13~26	0.352394	0.378356	0.217734	0.240918	0.729134	0.986311	-0.34898
13~27	0.234737	0.217339	0.348807	0.415181	0.889178	0.995741	0.140335
13~28	0.291806	0.21173	0.320701	0.386414	0.489846	0.967027	0.697701
13~29	0.398751	0.415559	0.218516	0.230724	0.818951	0.99358	-0.23057
13~30	0.367424	0.344431	0.469557	0.316728	0.862139	0.995741	0.174899
13~31	0.443637	0.426985	0.319728	0.312572	0.872206	0.995741	0.162007
13~32	0.499771	0.350478	0.294643	0.334235	0.151982	0.924237	1.463597
13~33	0.236114	0.287872	0.399717	0.278735	0.649736	0.972006	-0.45796
13~34	0.472819	0.518493	0.255778	0.271107	0.596442	0.967027	-0.53427
13~35	0.476087	0.418861	0.195315	0.249416	0.433779	0.964667	0.7916
13~36	0.288574	0.28843	0.318502	0.339914	0.99893	0.999007	0.001351
13~37	0.34477	0.315802	0.33144	0.297857	0.779458	0.992706	0.282132
13~38	0.305186	0.286616	0.355207	0.333356	0.869367	0.995741	0.16564
13~39	0.38273	0.356036	0.286077	0.254851	0.764149	0.986311	0.302318
13~40	0.364091	0.36622	0.320514	0.320601	0.983802	0.998253	-0.02044
13~41	0.394631	0.340808	0.278534	0.282819	0.558572	0.967027	0.590458
13~42	0.48909	0.474282	0.321226	0.312263	0.886487	0.995741	0.143766
13~43	0.436635	0.379068	0.370082	0.236337	0.567847	0.967027	0.577078

13~44	0.403658	0.394825	0.270097	0.405163	0.936822	0.997978	0.07982
13~45	0.373309	0.37728	0.269787	0.28091	0.964807	0.998253	-0.04443
13~46	0.493076	0.445229	0.251283	0.290442	0.589468	0.967027	0.544481
13~47	0.352181	0.493332	0.240022	0.22415	0.070016	0.924237	-1.86732
13~48	0.451091	0.325845	0.385838	0.267089	0.257492	0.924237	1.150576
13~49	0.445027	0.340909	0.263478	0.252213	0.222604	0.924237	1.241076
13~50	0.725506	0.682001	0.270333	0.273671	0.625387	0.967027	0.492456
13~51	0.504836	0.434772	0.490561	0.447656	0.649659	0.972006	0.458065
13~52	0.425822	0.391991	0.45032	0.332758	0.795673	0.993329	0.260879
13~53	0.43274	0.407626	0.359471	0.335655	0.825685	0.994197	0.221849
13~54	0.581364	0.456889	0.374433	0.33489	0.289408	0.924237	1.075271
13~55	0.40931	0.314182	0.284085	0.397707	0.39823	0.964667	0.854955
14~15	0.629814	0.566251	0.208813	0.243552	0.392166	0.964667	0.86611
14~16	0.481595	0.298626	0.313636	0.426025	0.137732	0.924237	1.518066
14~17	0.513755	0.456866	0.300281	0.280044	0.550997	0.967027	0.601925
14~18	0.414929	0.244604	0.274636	0.235201	0.04856	0.924237	2.041716
14~19	0.357931	0.235856	0.27833	0.256696	0.169989	0.924237	1.400282
14~20	0.469046	0.384292	0.327512	0.233709	0.369526	0.964667	0.908749
14~21	0.486237	0.402945	0.234954	0.231944	0.279604	0.924237	1.097745
14~22	0.511774	0.392937	0.294762	0.219687	0.171248	0.924237	1.396056
14~23	0.473572	0.430838	0.28221	0.232927	0.616151	0.967027	0.505699
14~24	0.417399	0.356158	0.272827	0.189299	0.431785	0.964667	0.795067
14~25	0.492939	0.46058	0.288109	0.202288	0.694155	0.984023	0.396393
14~26	0.365897	0.377601	0.270292	0.251474	0.891228	0.995741	-0.13772
14~27	0.58695	0.46237	0.305757	0.255285	0.183946	0.924237	1.354728
14~28	0.49494	0.405298	0.283508	0.293686	0.345027	0.957459	0.956846
14~29	0.373362	0.360343	0.376325	0.287053	0.904719	0.995741	0.12057
14~30	0.458313	0.428686	0.370755	0.306927	0.791333	0.993329	0.266555
14~31	0.386689	0.354604	0.429538	0.415851	0.816777	0.993329	0.233394
14~32	0.355371	0.451428	0.450744	0.259713	0.433096	0.964667	-0.79279
14~33	0.43183	0.300851	0.33899	0.295808	0.214894	0.924237	1.262477
14~34	0.465803	0.382094	0.316455	0.330461	0.430488	0.964667	0.797329
14~35	0.460668	0.409982	0.262532	0.24326	0.542333	0.967027	0.615138
14~36	0.402277	0.30286	0.34661	0.412799	0.425118	0.964667	0.806733
14~37	0.383181	0.354994	0.3826	0.269311	0.79291	0.993329	0.264599
14~38	0.540925	0.347281	0.314487	0.404188	0.106157	0.924237	1.657264
14~39	0.404233	0.329053	0.342142	0.265109	0.457608	0.964667	0.750876
14~40	0.491919	0.322862	0.254448	0.472555	0.188359	0.924237	1.351676
14~41	0.426242	0.391964	0.345856	0.289486	0.741554	0.986311	0.33236
14~42	0.505983	0.367925	0.338458	0.341716	0.219437	0.924237	1.2498
14~43	0.432326	0.342325	0.413727	0.317817	0.460747	0.964948	0.745604
14~44	0.48449	0.426501	0.318687	0.313984	0.576267	0.967027	0.563977
14~45	0.433447	0.302351	0.299043	0.333495	0.209499	0.924237	1.277799
14~46	0.493914	0.357734	0.326732	0.310261	0.197256	0.924237	1.313706

14~47	0.436855	0.348822	0.284728	0.333265	0.385753	0.964667	0.878025
14~48	0.569173	0.353321	0.24766	0.263682	0.013376	0.924237	2.601816
14~49	0.457838	0.323421	0.277879	0.242761	0.122895	0.924237	1.579823
14~50	0.465611	0.450461	0.420363	0.423868	0.912627	0.995741	0.1105
14~51	0.531366	0.529733	0.321766	0.246602	0.986205	0.998253	0.017411
14~52	0.543498	0.355163	0.450722	0.431369	0.197684	0.924237	1.312423
14~53	0.530524	0.386169	0.505588	0.513593	0.388847	0.964667	0.87226
14~54	0.404049	0.382376	0.446674	0.288727	0.861759	0.995741	0.175386
14~55	0.421336	0.497034	0.311723	0.253366	0.420071	0.964667	-0.81564
15~16	0.30833	0.202434	0.311372	0.443493	0.396144	0.964667	0.85878
15~17	0.362965	0.380783	0.265807	0.321525	0.852789	0.995741	-0.1869
15~18	0.408851	0.27953	0.289094	0.225782	0.136148	0.924237	1.524392
15~19	0.370416	0.266649	0.290288	0.24247	0.242482	0.924237	1.188335
15~20	0.422853	0.372157	0.288277	0.249639	0.567934	0.967027	0.576397
15~21	0.409626	0.403327	0.227917	0.235652	0.933744	0.997713	0.083719
15~22	0.443611	0.41703	0.289168	0.229493	0.757243	0.986311	0.311467
15~23	0.432596	0.435982	0.251425	0.212171	0.96468	0.998253	-0.04459
15~24	0.46693	0.394775	0.27656	0.223092	0.385347	0.964667	0.878782
15~25	0.471587	0.439412	0.262103	0.207876	0.679854	0.98051	0.416036
15~26	0.469408	0.51614	0.30129	0.207232	0.585156	0.967027	-0.55082
15~27	0.397188	0.356956	0.344705	0.320429	0.712553	0.986311	0.37135
15~28	0.615158	0.587672	0.221041	0.262601	0.728215	0.986311	0.35022
15~29	0.33842	0.352899	0.325597	0.262485	0.881749	0.995741	-0.14981
15~30	0.338576	0.390106	0.283618	0.295533	0.586918	0.967027	-0.54823
15~31	0.320506	0.244408	0.308875	0.413744	0.521945	0.967027	0.646674
15~32	0.32172	0.385058	0.314203	0.257749	0.504149	0.967027	-0.67474
15~33	0.340214	0.229736	0.25877	0.252993	0.192538	0.924237	1.327995
15~34	0.464666	0.320613	0.248297	0.368495	0.162449	0.924237	1.42612
15~35	0.390047	0.362505	0.231354	0.263168	0.733321	0.986311	0.343365
15~36	0.340925	0.202451	0.26711	0.376948	0.196203	0.924237	1.316873
15~37	0.287829	0.294866	0.327213	0.28468	0.944302	0.998253	-0.07035
15~38	0.382087	0.183289	0.321774	0.372066	0.085834	0.924237	1.766249
15~39	0.358278	0.274213	0.333474	0.236483	0.380982	0.964667	0.886972
15~40	0.358953	0.257439	0.232061	0.394338	0.334097	0.957459	0.979034
15~41	0.357019	0.308855	0.31541	0.280787	0.62369	0.967027	0.494883
15~42	0.389899	0.32665	0.300029	0.362852	0.560319	0.967027	0.587826
15~43	0.359866	0.30403	0.302805	0.312826	0.579795	0.967027	0.558744
15~44	0.393563	0.413727	0.308684	0.290416	0.837379	0.99525	-0.20674
15~45	0.356153	0.24716	0.289073	0.333484	0.287652	0.924237	1.079255
15~46	0.447696	0.337275	0.333526	0.308004	0.297828	0.928415	1.056391
15~47	0.424473	0.392691	0.29716	0.307021	0.747762	0.986311	0.324068
15~48	0.430098	0.290722	0.230197	0.249478	0.081667	0.924237	1.791231
15~49	0.387736	0.271395	0.268476	0.279124	0.198821	0.924237	1.309024
15~50	0.364817	0.324839	0.349555	0.446516	0.759149	0.986311	0.308939

15~51	0.387365	0.441829	0.283747	0.278229	0.554741	0.967027	-0.59625
15~52	0.380248	0.225015	0.397203	0.366513	0.220284	0.924237	1.247457
15~53	0.404764	0.248772	0.351879	0.421647	0.222055	0.924237	1.242581
15~54	0.345496	0.299662	0.303846	0.258321	0.621483	0.967027	0.498042
15~55	0.319208	0.392897	0.278304	0.259647	0.405836	0.964667	-0.84112
16~17	0.693645	0.510057	0.413552	0.591416	0.270947	0.924237	1.118061
16~18	0.276701	0.2286	0.303137	0.243822	0.595913	0.967027	0.535039
16~19	0.262598	0.261353	0.308747	0.327882	0.990449	0.998253	0.012054
16~20	0.413699	0.4468	0.339179	0.279155	0.746116	0.986311	-0.32626
16~21	0.322073	0.366005	0.341132	0.267696	0.663926	0.973774	-0.43811
16~22	0.418756	0.401717	0.323083	0.28133	0.864037	0.995741	0.172467
16~23	0.38984	0.399235	0.318089	0.295101	0.925589	0.997713	-0.09405
16~24	0.254827	0.243697	0.27569	0.245964	0.896726	0.995741	0.130718
16~25	0.391646	0.367676	0.30793	0.280636	0.804164	0.993329	0.249798
16~26	0.273153	0.230621	0.250787	0.275144	0.621112	0.967027	0.498575
16~27	0.385341	0.226166	0.356609	0.400495	0.203148	0.924237	1.29622
16~28	0.27997	0.116526	0.393944	0.417021	0.222218	0.924237	1.242136
16~29	0.230746	0.30681	0.492999	0.260746	0.56246	0.967027	-0.5846
16~30	0.573716	0.509715	0.497078	0.372968	0.659093	0.972006	0.444848
16~31	0.306273	0.53968	0.48039	0.341404	0.096175	0.924237	-1.70838
16~32	0.424117	0.508669	0.592569	0.449659	0.626328	0.967027	-0.49111
16~33	0.364854	0.374308	0.376171	0.320239	0.93436	0.997713	-0.08294
16~34	0.349129	0.40147	0.330658	0.303757	0.615785	0.967027	-0.50623
16~35	0.374379	0.31242	0.351013	0.310922	0.570048	0.967027	0.573238
16~36	0.349393	0.355417	0.341814	0.314675	0.955411	0.998253	-0.0563
16~37	0.346569	0.278155	0.429081	0.262872	0.553398	0.967027	0.599
16~38	0.348306	0.347149	0.3595	0.288309	0.991397	0.998253	0.010858
16~39	0.260138	0.294159	0.421441	0.252639	0.767789	0.986311	-0.29751
16~40	0.430364	0.381507	0.342822	0.33777	0.661337	0.973326	0.441716
16~41	0.382438	0.323903	0.389799	0.295365	0.608242	0.967027	0.517111
16~42	0.485446	0.533137	0.461999	0.336411	0.720816	0.986311	-0.36018
16~43	0.552068	0.556623	0.565765	0.279146	0.974738	0.998253	-0.03195
16~44	0.384289	0.299023	0.341281	0.529749	0.554997	0.967027	0.59586
16~45	0.372958	0.401297	0.349858	0.302033	0.7919	0.993329	-0.26581
16~46	0.365289	0.387968	0.505271	0.297456	0.865669	0.995741	-0.17056
16~47	0.306365	0.38559	0.305603	0.310987	0.433947	0.964667	-0.79131
16~48	0.482762	0.336806	0.34906	0.315441	0.186519	0.924237	1.346625
16~49	0.420871	0.298523	0.339367	0.27574	0.23376	0.924237	1.211068
16~50	0.460398	0.492544	0.54753	0.364996	0.834534	0.994675	-0.21041
16~51	0.564986	0.456851	0.512637	0.483875	0.509247	0.967027	0.666648
16~52	0.569599	0.422806	0.399121	0.3394	0.232582	0.924237	1.214186
16~53	0.380571	0.395509	0.510627	0.335646	0.916754	0.995741	-0.10526
16~54	0.580673	0.507288	0.503104	0.309219	0.596439	0.967027	0.534269
16~55	0.492275	0.364526	0.345817	0.435275	0.320841	0.945335	1.006609

17~18	0.364254	0.26737	0.255318	0.222885	0.223111	0.924237	1.239688
17~19	0.363245	0.207097	0.267894	0.33965	0.122511	0.924237	1.5815
17~20	0.522127	0.3766	0.251537	0.420139	0.198183	0.924237	1.310928
17~21	0.424978	0.372794	0.302022	0.278091	0.584343	0.967027	0.552024
17~22	0.530425	0.431192	0.239433	0.261734	0.230138	0.924237	1.220691
17~23	0.492255	0.427285	0.243649	0.27176	0.442135	0.964667	0.777172
17~24	0.271816	0.308874	0.252835	0.208357	0.627273	0.967027	-0.48976
17~25	0.461519	0.419451	0.257289	0.245311	0.610094	0.967027	0.514432
17~26	0.294222	0.275476	0.212472	0.274166	0.814076	0.993329	0.236901
17~27	0.313672	0.308825	0.325215	0.306452	0.96266	0.998253	0.047143
17~28	0.258606	0.27579	0.313272	0.304319	0.865081	0.995741	-0.17113
17~29	0.357957	0.316384	0.261932	0.256151	0.624567	0.967027	0.493628
17~30	0.556332	0.53689	0.371981	0.33561	0.867182	0.995741	0.168437
17~31	0.408811	0.439357	0.46412	0.480223	0.843167	0.995741	-0.19928
17~32	0.477889	0.676128	0.591851	0.30757	0.199172	0.924237	-1.31373
17~33	0.399091	0.340039	0.369827	0.371033	0.626609	0.967027	0.490711
17~34	0.457964	0.336519	0.278335	0.389826	0.27278	0.924237	1.11372
17~35	0.477471	0.350961	0.285019	0.300686	0.191511	0.924237	1.331144
17~36	0.350283	0.262219	0.359235	0.412012	0.486	0.967027	0.703938
17~37	0.352081	0.304349	0.381037	0.276531	0.659131	0.972006	0.444952
17~38	0.363288	0.212686	0.375792	0.395154	0.236511	0.924237	1.203832
17~39	0.372322	0.308656	0.255167	0.256386	0.448532	0.964667	0.766234
17~40	0.479361	0.333305	0.331909	0.420802	0.24029	0.924237	1.193991
17~41	0.430031	0.344878	0.319444	0.310064	0.410944	0.964667	0.831911
17~42	0.522803	0.45377	0.424877	0.411844	0.614981	0.967027	0.507383
17~43	0.522025	0.419851	0.442364	0.411306	0.467213	0.967027	0.734813
17~44	0.400498	0.475699	0.341624	0.322368	0.491038	0.967027	-0.69577
17~45	0.41044	0.303137	0.314165	0.398003	0.360024	0.964667	0.927148
17~46	0.484267	0.387984	0.44183	0.347762	0.463764	0.965903	0.740559
17~47	0.4008	0.333961	0.261971	0.36612	0.518459	0.967027	0.652132
17~48	0.571675	0.372947	0.272836	0.301903	0.03992	0.924237	2.131786
17~49	0.487233	0.311164	0.283686	0.288414	0.066101	0.924237	1.895329
17~50	0.586783	0.458393	0.404295	0.496512	0.385885	0.964667	0.877777
17~51	0.574262	0.627671	0.50281	0.323475	0.702913	0.9857	-0.38444
17~52	0.612917	0.324945	0.306768	0.424152	0.020904	0.924237	2.415743
17~53	0.399228	0.304322	0.488669	0.444638	0.53679	0.967027	0.62365
17~54	0.620302	0.538921	0.490442	0.324693	0.555016	0.967027	0.595832
17~55	0.526065	0.529277	0.338323	0.302488	0.975668	0.998253	-0.03071
18~19	0.707232	0.591202	0.196383	0.251863	0.120078	0.924237	1.592225
18~20	0.696508	0.610859	0.240694	0.273135	0.310958	0.936999	1.027675
18~21	0.536324	0.487243	0.241996	0.316524	0.592414	0.967027	0.540158
18~22	0.578738	0.455556	0.245937	0.278712	0.156401	0.924237	1.447534
18~23	0.528093	0.50974	0.256831	0.291759	0.837753	0.99525	0.206257
18~24	0.592186	0.519502	0.215465	0.285496	0.378655	0.964667	0.89136

18~25	0.599166	0.525167	0.232433	0.325593	0.421817	0.964667	0.81255
18~26	0.468923	0.397791	0.224743	0.29873	0.40939	0.964667	0.834704
18~27	0.357157	0.22799	0.289518	0.237937	0.144324	0.924237	1.492333
18~28	0.355229	0.300859	0.308598	0.264004	0.565357	0.967027	0.580255
18~29	0.409375	0.318866	0.242939	0.227174	0.244835	0.924237	1.182304
18~30	0.317924	0.206795	0.278403	0.265763	0.217495	0.924237	1.255194
18~31	0.372571	0.248324	0.274661	0.247972	0.153666	0.924237	1.457431
18~32	0.350068	0.260689	0.2752	0.199796	0.264167	0.924237	1.134297
18~33	0.320032	0.220671	0.292911	0.222063	0.250512	0.924237	1.167929
18~34	0.532991	0.463303	0.296203	0.297356	0.474465	0.967027	0.722813
18~35	0.486295	0.385336	0.24294	0.300973	0.260623	0.924237	1.142903
18~36	0.347855	0.176834	0.216169	0.2188	0.020641	0.924237	2.421129
18~37	0.362268	0.255174	0.319179	0.272246	0.275906	0.924237	1.106367
18~38	0.341141	0.188232	0.253767	0.181066	0.041324	0.924237	2.116045
18~39	0.418413	0.27084	0.250204	0.230687	0.06776	0.924237	1.883287
18~40	0.337308	0.184947	0.22411	0.234229	0.047871	0.924237	2.048358
18~41	0.419778	0.274104	0.273658	0.306627	0.13043	0.924237	1.54775
18~42	0.41363	0.29353	0.25297	0.222676	0.130907	0.924237	1.54577
18~43	0.322028	0.19535	0.273523	0.260116	0.153287	0.924237	1.458814
18~44	0.421672	0.354916	0.269717	0.251757	0.43697	0.964667	0.786071
18~45	0.411695	0.274401	0.249057	0.273399	0.113938	0.924237	1.620103
18~46	0.436646	0.336728	0.265723	0.230038	0.225719	0.924237	1.232587
18~47	0.497963	0.42126	0.184564	0.224757	0.255983	0.924237	1.154299
18~48	0.467033	0.208015	0.263939	0.279099	0.005708	0.924237	2.939645
18~49	0.479604	0.291373	0.248573	0.292561	0.038869	0.924237	2.143889
18~50	0.357951	0.255839	0.247555	0.200697	0.17404	0.924237	1.386768
18~51	0.326483	0.232412	0.302152	0.205586	0.265879	0.924237	1.131429
18~52	0.367309	0.187792	0.246159	0.239524	0.029069	0.924237	2.273382
18~53	0.310631	0.144368	0.306666	0.229874	0.065389	0.924237	1.902426
18~54	0.288138	0.222487	0.300007	0.197572	0.436468	0.964667	0.786939
18~55	0.314275	0.215222	0.248832	0.225248	0.208361	0.924237	1.281068
19~20	0.598904	0.572334	0.290047	0.253431	0.766534	0.986311	0.299166
19~21	0.430817	0.389566	0.255928	0.311683	0.657081	0.972006	0.44766
19~22	0.493728	0.397583	0.257739	0.315798	0.308745	0.936999	1.032456
19~23	0.448606	0.459274	0.255388	0.319785	0.909742	0.995741	-0.11416
19~24	0.51395	0.455542	0.216126	0.290932	0.484	0.967027	0.707193
19~25	0.487212	0.443879	0.252624	0.302717	0.633572	0.967027	0.480794
19~26	0.483732	0.397791	0.232348	0.332013	0.367511	0.964667	0.914926
19~27	0.294606	0.218925	0.266439	0.253697	0.377149	0.964667	0.894211
19~28	0.354589	0.286351	0.339995	0.289425	0.511996	0.967027	0.662301
19~29	0.36275	0.306421	0.241536	0.26348	0.496091	0.967027	0.687632
19~30	0.256919	0.198057	0.320101	0.301371	0.564322	0.967027	0.581808
19~31	0.330521	0.206569	0.25819	0.317623	0.193296	0.924237	1.325681
19~32	0.323751	0.20996	0.301332	0.275397	0.234038	0.924237	1.210332

19~33	0.285098	0.177723	0.270473	0.299934	0.253449	0.924237	1.160586
19~34	0.539548	0.442329	0.297196	0.33188	0.347038	0.957459	0.952815
19~35	0.424752	0.400208	0.291787	0.292516	0.797415	0.993329	0.258602
19~36	0.314151	0.17243	0.234148	0.249935	0.079521	0.924237	1.804519
19~37	0.31229	0.229921	0.323858	0.316617	0.434047	0.964667	0.791134
19~38	0.264009	0.151978	0.288708	0.256233	0.216075	0.924237	1.259162
19~39	0.348653	0.256725	0.288772	0.241595	0.297275	0.928415	1.057619
19~40	0.314836	0.161488	0.241594	0.286186	0.081766	0.924237	1.790627
19~41	0.330125	0.279602	0.321254	0.293727	0.617351	0.967027	0.503973
19~42	0.404327	0.271342	0.254691	0.278336	0.132767	0.924237	1.538105
19~43	0.304311	0.216144	0.289494	0.314513	0.374166	0.964667	0.899876
19~44	0.36359	0.309515	0.29557	0.355372	0.611891	0.967027	0.511836
19~45	0.346625	0.251101	0.274945	0.278085	0.294584	0.928415	1.06362
19~46	0.450235	0.361612	0.286066	0.319099	0.372589	0.964667	0.902885
19~47	0.533126	0.492827	0.257488	0.258426	0.633502	0.967027	0.480894
19~48	0.419713	0.267405	0.27446	0.267255	0.092314	0.924237	1.729352
19~49	0.440667	0.259178	0.257195	0.333502	0.066946	0.924237	1.889163
19~50	0.333618	0.258595	0.256346	0.259865	0.376744	0.964667	0.894978
19~51	0.283635	0.243004	0.304964	0.237167	0.652055	0.972006	0.4547
19~52	0.354291	0.174576	0.257233	0.215646	0.026167	0.924237	2.319288
19~53	0.288157	0.140359	0.294452	0.300409	0.134695	0.924237	1.530251
19~54	0.292637	0.221735	0.296654	0.301574	0.47017	0.967027	0.729907
19~55	0.28699	0.17894	0.260159	0.2213	0.178882	0.924237	1.370934
20~21	0.615386	0.616222	0.262118	0.245426	0.991992	0.998253	-0.01011
20~22	0.750046	0.696676	0.198141	0.234405	0.451962	0.964667	0.760408
20~23	0.675762	0.680394	0.21473	0.257696	0.95217	0.998253	-0.0604
20~24	0.530989	0.534803	0.217481	0.24229	0.95949	0.998253	-0.05115
20~25	0.715871	0.672164	0.216171	0.238611	0.557212	0.967027	0.592511
20~26	0.44188	0.446827	0.248853	0.274433	0.953841	0.998253	-0.05829
20~27	0.381668	0.26441	0.333931	0.276405	0.249137	0.924237	1.171389
20~28	0.344608	0.269596	0.302397	0.305284	0.452159	0.964667	0.760076
20~29	0.479683	0.41346	0.266969	0.260588	0.445064	0.964667	0.772153
20~30	0.423661	0.40649	0.320058	0.208194	0.844358	0.995741	0.197877
20~31	0.493593	0.387343	0.263332	0.281697	0.23736	0.924237	1.201611
20~32	0.480389	0.418857	0.282134	0.220431	0.462241	0.964948	0.743104
20~33	0.396701	0.345346	0.330197	0.290007	0.615353	0.967027	0.506847
20~34	0.642487	0.586543	0.242204	0.275635	0.50963	0.967027	0.666041
20~35	0.606897	0.515077	0.216246	0.305797	0.288569	0.924237	1.077171
20~36	0.437672	0.308598	0.232382	0.241902	0.102242	0.924237	1.676817
20~37	0.439967	0.35773	0.358643	0.27258	0.435392	0.964667	0.788802
20~38	0.388435	0.28083	0.334533	0.215891	0.242831	0.924237	1.189374
20~39	0.476843	0.371595	0.289977	0.247659	0.239448	0.924237	1.196173
20~40	0.46836	0.304203	0.240639	0.245941	0.044907	0.924237	2.077927
20~41	0.480132	0.393698	0.328374	0.284002	0.393771	0.964667	0.863146

20~42	0.540507	0.46922	0.275073	0.244077	0.405894	0.964667	0.84101
20~43	0.456014	0.394206	0.329042	0.214449	0.493534	0.967027	0.692403
20~44	0.48562	0.435355	0.29217	0.37825	0.647554	0.972006	0.461025
20~45	0.513603	0.430023	0.285275	0.266181	0.358231	0.964667	0.930655
20~46	0.538081	0.442093	0.243113	0.268219	0.254749	0.924237	1.157355
20~47	0.515531	0.467132	0.211295	0.235941	0.508893	0.967027	0.667209
20~48	0.593531	0.372706	0.228434	0.255373	0.007879	0.924237	2.814085
20~49	0.593453	0.39177	0.232901	0.34125	0.043913	0.924237	2.104788
20~50	0.483253	0.420299	0.243528	0.258527	0.444667	0.964667	0.772831
20~51	0.455748	0.413115	0.297062	0.239629	0.631744	0.967027	0.483392
20~52	0.490601	0.326328	0.275682	0.233605	0.056581	0.924237	1.969893
20~53	0.397321	0.289325	0.330844	0.304862	0.304109	0.933062	1.042547
20~54	0.415448	0.391435	0.294527	0.252945	0.790153	0.993329	0.2681
20~55	0.428935	0.354492	0.281049	0.204354	0.361318	0.964667	0.924624
21~22	0.717722	0.733282	0.247898	0.140162	0.81592	0.993329	-0.23451
21~23	0.670352	0.7368	0.307349	0.139178	0.390578	0.964667	-0.87256
21~24	0.501174	0.573432	0.266741	0.165968	0.329207	0.957459	-0.98912
21~25	0.588217	0.655335	0.287184	0.164917	0.378179	0.964667	-0.89412
21~26	0.402488	0.407595	0.27664	0.233566	0.951608	0.998253	-0.06111
21~27	0.398048	0.308427	0.284003	0.297778	0.348812	0.957459	0.949272
21~28	0.365343	0.311796	0.285856	0.250109	0.544767	0.967027	0.611415
21~29	0.419454	0.428997	0.26381	0.215341	0.904119	0.995741	-0.12131
21~30	0.375386	0.406729	0.323427	0.220239	0.726981	0.986311	-0.35207
21~31	0.422735	0.373248	0.295408	0.284295	0.602903	0.967027	0.524853
21~32	0.409128	0.405595	0.30669	0.196243	0.966192	0.998253	0.042711
21~33	0.377598	0.306294	0.309833	0.27185	0.457954	0.964667	0.750293
21~34	0.522204	0.552825	0.259388	0.244369	0.71104	0.986311	-0.3734
21~35	0.514898	0.483076	0.239401	0.249654	0.690846	0.984023	0.400923
21~36	0.388771	0.312627	0.263389	0.223139	0.345554	0.957459	0.955788
21~37	0.396587	0.387065	0.345346	0.293433	0.927948	0.997713	0.091062
21~38	0.425355	0.296267	0.281899	0.266451	0.156736	0.924237	1.44633
21~39	0.406273	0.424403	0.269134	0.233527	0.826645	0.994197	-0.22061
21~40	0.445186	0.313096	0.235813	0.235482	0.093059	0.924237	1.725249
21~41	0.43193	0.419159	0.329526	0.252415	0.894961	0.995741	0.132966
21~42	0.447725	0.426576	0.290997	0.196579	0.792823	0.993329	0.264751
21~43	0.403605	0.370935	0.337119	0.239783	0.730893	0.986311	0.346759
21~44	0.416374	0.450996	0.308073	0.267536	0.715089	0.986311	-0.36792
21~45	0.432718	0.355234	0.282848	0.277659	0.400661	0.964667	0.850513
21~46	0.437644	0.353139	0.269374	0.315689	0.379234	0.964667	0.890266
21~47	0.429128	0.439338	0.240721	0.257278	0.900136	0.995741	-0.12638
21~48	0.482805	0.334972	0.226043	0.279554	0.080177	0.924237	1.800429
21~49	0.496703	0.348366	0.265575	0.303324	0.116683	0.924237	1.607492
21~50	0.404847	0.405852	0.299438	0.270952	0.991438	0.998253	-0.01081
21~51	0.389129	0.384649	0.311835	0.225937	0.960235	0.998253	0.050207

21~52	0.428912	0.322896	0.294559	0.207864	0.205208	0.924237	1.291483
21~53	0.350772	0.307254	0.338205	0.279016	0.66992	0.975425	0.429776
21~54	0.331382	0.363152	0.355397	0.250547	0.750254	0.986311	-0.32088
21~55	0.410638	0.331976	0.285018	0.209273	0.343267	0.957459	0.960386
22~23	0.794722	0.822473	0.158875	0.083588	0.51185	0.967027	-0.66253
22~24	0.511739	0.553525	0.232563	0.227937	0.580128	0.967027	-0.55825
22~25	0.682186	0.71578	0.240974	0.109722	0.590829	0.967027	-0.54248
22~26	0.418345	0.425345	0.230109	0.278914	0.932952	0.997713	-0.08472
22~27	0.397212	0.2583	0.347429	0.293161	0.193861	0.924237	1.323963
22~28	0.392783	0.275705	0.30964	0.243837	0.207097	0.924237	1.284717
22~29	0.462567	0.457976	0.249625	0.230141	0.953493	0.998253	0.058729
22~30	0.445234	0.441412	0.325123	0.205106	0.965352	0.998253	0.043776
22~31	0.466651	0.402574	0.304743	0.273536	0.501423	0.967027	0.679091
22~32	0.487739	0.454642	0.290416	0.176857	0.670885	0.975425	0.428899
22~33	0.43075	0.307783	0.319615	0.280016	0.217542	0.924237	1.255063
22~34	0.624921	0.573424	0.209368	0.244586	0.488897	0.967027	0.699238
22~35	0.587738	0.529755	0.22786	0.266272	0.474171	0.967027	0.723296
22~36	0.424685	0.303184	0.285903	0.232495	0.162157	0.924237	1.427137
22~37	0.409447	0.398597	0.357909	0.318049	0.922219	0.997713	0.098326
22~38	0.410594	0.257338	0.36379	0.258938	0.147426	0.924237	1.480551
22~39	0.433621	0.423753	0.301114	0.264543	0.915545	0.995741	0.106795
22~40	0.483615	0.308575	0.228769	0.248006	0.029747	0.924237	2.26325
22~41	0.50322	0.444071	0.279045	0.288958	0.525223	0.967027	0.64156
22~42	0.528032	0.458054	0.275236	0.226657	0.401052	0.964667	0.849801
22~43	0.461129	0.406579	0.349192	0.228936	0.569357	0.967027	0.574742
22~44	0.46078	0.442567	0.307328	0.273936	0.84885	0.995741	0.191962
22~45	0.485285	0.383109	0.292597	0.24982	0.257319	0.924237	1.151003
22~46	0.515588	0.40867	0.249822	0.284179	0.225055	0.924237	1.234389
22~47	0.496977	0.453606	0.218111	0.249665	0.571143	0.967027	0.571602
22~48	0.573631	0.416516	0.223564	0.293646	0.070075	0.924237	1.866908
22~49	0.559444	0.436692	0.24423	0.307562	0.179434	0.924237	1.36915
22~50	0.472978	0.469037	0.291504	0.24486	0.964483	0.998253	0.044839
22~51	0.461276	0.464177	0.308626	0.233491	0.974346	0.998253	-0.03238
22~52	0.510654	0.341234	0.270501	0.231361	0.046386	0.924237	2.062958
22~53	0.391129	0.351813	0.364664	0.291156	0.717613	0.986311	0.364506
22~54	0.417517	0.455417	0.334883	0.2168	0.678617	0.98051	-0.41807
22~55	0.421353	0.332755	0.293059	0.200803	0.280826	0.924237	1.096036
23~24	0.506273	0.604578	0.231624	0.182298	0.157852	0.924237	-1.44234
23~25	0.698284	0.791618	0.216098	0.086662	0.087154	0.924237	-1.7791
23~26	0.429069	0.453758	0.226678	0.247977	0.750313	0.986311	-0.32067
23~27	0.400227	0.319091	0.339894	0.297415	0.441009	0.964667	0.779107
23~28	0.386944	0.324505	0.29364	0.236291	0.478088	0.967027	0.716856
23~29	0.422737	0.488725	0.324745	0.252523	0.492442	0.967027	-0.69351
23~30	0.42785	0.454633	0.327654	0.210439	0.764021	0.986311	-0.30272

23~31	0.438883	0.4307	0.307218	0.264667	0.93078	0.997713	0.087474
23~32	0.468636	0.438743	0.277518	0.162799	0.684816	0.983461	0.409711
23~33	0.428072	0.324093	0.299332	0.284857	0.281293	0.924237	1.093833
23~34	0.580153	0.598975	0.211712	0.249612	0.802882	0.993329	-0.25147
23~35	0.540926	0.550275	0.221316	0.282542	0.909751	0.995741	-0.11415
23~36	0.44573	0.344666	0.269234	0.276978	0.261905	0.924237	1.139781
23~37	0.394144	0.433857	0.39249	0.363551	0.748988	0.986311	-0.32244
23~38	0.410268	0.314961	0.368797	0.280066	0.379772	0.964667	0.889251
23~39	0.441673	0.465774	0.3233	0.284289	0.809525	0.993329	-0.24282
23~40	0.474924	0.360664	0.266046	0.293021	0.215771	0.924237	1.260016
23~41	0.462142	0.474905	0.322788	0.305575	0.901381	0.995741	-0.12479
23~42	0.495626	0.448975	0.284224	0.210903	0.572764	0.967027	0.569186
23~43	0.445483	0.409081	0.341384	0.23858	0.703417	0.9857	0.383934
23~44	0.469281	0.480737	0.314657	0.26966	0.905284	0.995741	-0.11983
23~45	0.461218	0.385847	0.301959	0.267107	0.422666	0.964667	0.811051
23~46	0.47892	0.412878	0.254121	0.270122	0.442556	0.964667	0.776449
23~47	0.466924	0.479322	0.219054	0.203755	0.858139	0.995741	-0.18003
23~48	0.539817	0.428838	0.217984	0.306942	0.203539	0.924237	1.295075
23~49	0.545481	0.44582	0.242338	0.299807	0.265151	0.924237	1.131923
23~50	0.454175	0.483491	0.298669	0.217508	0.733988	0.986311	-0.34247
23~51	0.450699	0.467992	0.29161	0.195892	0.829965	0.9943	-0.21643
23~52	0.495436	0.361132	0.280284	0.248118	0.128333	0.924237	1.556519
23~53	0.388057	0.338873	0.343562	0.31539	0.649729	0.972006	0.457966
23~54	0.411103	0.465526	0.319075	0.210382	0.535369	0.967027	-0.62636
23~55	0.417608	0.359654	0.282833	0.217903	0.487443	0.967027	0.701595
24~25	0.676856	0.671571	0.163255	0.196845	0.928445	0.997713	0.090432
24~26	0.661875	0.668632	0.137552	0.170103	0.893182	0.995741	-0.13523
24~27	0.430724	0.313033	0.29047	0.336371	0.254738	0.924237	1.157383
24~28	0.44452	0.389463	0.341945	0.259511	0.582896	0.967027	0.55416
24~29	0.447357	0.42617	0.280703	0.28014	0.817437	0.993329	0.232538
24~30	0.293414	0.303683	0.285911	0.212146	0.901565	0.995741	-0.12456
24~31	0.32004	0.314241	0.282449	0.221129	0.944662	0.998253	0.069898
24~32	0.280427	0.310054	0.271173	0.180052	0.697374	0.984674	-0.39199
24~33	0.323033	0.241279	0.278652	0.241003	0.342425	0.957459	0.962085
24~34	0.472093	0.535651	0.23557	0.206026	0.384288	0.964667	-0.88076
24~35	0.429383	0.419671	0.240593	0.288734	0.910624	0.995741	0.113043
24~36	0.367334	0.27136	0.23369	0.248516	0.227888	0.924237	1.226728
24~37	0.361187	0.344044	0.314152	0.256911	0.855926	0.995741	0.18287
24~38	0.355199	0.237311	0.3109	0.25263	0.210922	0.924237	1.27373
24~39	0.406585	0.349765	0.294928	0.280104	0.547529	0.967027	0.6072
24~40	0.325451	0.301727	0.241751	0.221392	0.755138	0.986311	0.314259
24~41	0.360063	0.351957	0.31266	0.229823	0.928636	0.997713	0.09019
24~42	0.340691	0.362887	0.238129	0.215769	0.766014	0.986311	-0.29985
24~43	0.323276	0.291452	0.28041	0.196376	0.690895	0.984023	0.400856

24~44	0.40083	0.402134	0.270419	0.225218	0.98729	0.998253	−0.01604
24~45	0.388491	0.309979	0.266204	0.223629	0.334459	0.957459	0.97829
24~46	0.389328	0.3256	0.240809	0.251652	0.430454	0.964667	0.797389
24~47	0.466861	0.465262	0.213601	0.227192	0.982287	0.998253	0.022357
24~48	0.390305	0.293453	0.214778	0.267554	0.224386	0.924237	1.23621
24~49	0.416579	0.330802	0.228923	0.266456	0.292916	0.927465	1.067357
24~50	0.289523	0.353905	0.254776	0.206309	0.40097	0.964667	−0.84995
24~51	0.27345	0.347477	0.284895	0.213496	0.375088	0.964667	−0.89812
24~52	0.350549	0.28946	0.26094	0.197366	0.425154	0.964667	0.806669
24~53	0.295029	0.303626	0.331375	0.226508	0.926962	0.997713	−0.09231
24~54	0.233273	0.283712	0.291793	0.22127	0.555502	0.967027	−0.5951
24~55	0.278138	0.29424	0.243327	0.21057	0.829472	0.9943	−0.21695
25~26	0.540557	0.507397	0.182464	0.277236	0.662738	0.973458	0.439762
25~27	0.421292	0.319684	0.325714	0.307841	0.331037	0.957459	0.985333
25~28	0.394108	0.327817	0.31428	0.275973	0.496203	0.967027	0.687453
25~29	0.475241	0.466891	0.322105	0.277934	0.932663	0.997713	0.085088
25~30	0.402669	0.443967	0.31271	0.202154	0.628744	0.967027	−0.48807
25~31	0.428304	0.394135	0.303347	0.27357	0.718677	0.986311	0.363068
25~32	0.413125	0.417548	0.295052	0.172901	0.954883	0.998253	−0.05703
25~33	0.388371	0.313271	0.309907	0.324239	0.470264	0.967027	0.729752
25~34	0.564742	0.61001	0.250222	0.215219	0.555857	0.967027	−0.59456
25~35	0.542474	0.546562	0.264044	0.305043	0.964925	0.998253	−0.04428
25~36	0.452671	0.333162	0.242568	0.287461	0.173196	0.924237	1.389565
25~37	0.436286	0.410167	0.361101	0.350003	0.822568	0.994197	0.225886
25~38	0.43243	0.289909	0.32988	0.30213	0.175038	0.924237	1.383478
25~39	0.475023	0.473369	0.293246	0.27686	0.985878	0.998253	0.017823
25~40	0.466875	0.349606	0.251851	0.284274	0.185901	0.924237	1.348562
25~41	0.438622	0.428912	0.356183	0.313423	0.929752	0.997713	0.088775
25~42	0.470464	0.469214	0.294796	0.208786	0.988173	0.998253	0.014927
25~43	0.441376	0.414574	0.313571	0.222291	0.76532	0.986311	0.30077
25~44	0.454626	0.494665	0.288945	0.25935	0.657173	0.972006	−0.44753
25~45	0.425091	0.399791	0.332171	0.273707	0.800538	0.993329	0.254525
25~46	0.49872	0.42286	0.232742	0.270464	0.358931	0.964667	0.929285
25~47	0.458073	0.483313	0.219259	0.226053	0.728991	0.986311	−0.34918
25~48	0.518356	0.405055	0.199945	0.246301	0.126672	0.924237	1.563553
25~49	0.524514	0.443492	0.235145	0.301151	0.358907	0.964667	0.929333
25~50	0.462075	0.494843	0.268588	0.214186	0.682314	0.980868	−0.41265
25~51	0.425079	0.463766	0.296951	0.193927	0.634459	0.967322	−0.47991
25~52	0.466846	0.380141	0.275615	0.248377	0.317202	0.942605	1.014314
25~53	0.387176	0.330493	0.347382	0.311475	0.601271	0.967027	0.527227
25~54	0.386838	0.439652	0.306866	0.224491	0.55252	0.967027	−0.59961
25~55	0.390188	0.371337	0.270953	0.178148	0.799749	0.993329	0.25572
26~27	0.376497	0.283967	0.28	0.285659	0.320426	0.945335	1.007485
26~28	0.491564	0.521456	0.386532	0.292668	0.791468	0.993329	−0.26638

26~29	0.408298	0.404714	0.27969	0.273044	0.968408	0.998253	0.039882
26~30	0.253503	0.264454	0.280194	0.308061	0.909264	0.995741	-0.11477
26~31	0.254625	0.267767	0.239853	0.294861	0.880541	0.995741	-0.15135
26~32	0.261355	0.288653	0.261796	0.254139	0.746757	0.986311	-0.32541
26~33	0.24206	0.207802	0.268578	0.268989	0.697141	0.984674	0.392311
26~34	0.456609	0.431454	0.249443	0.244273	0.755757	0.986311	0.313438
26~35	0.387864	0.345357	0.239292	0.302174	0.631938	0.967027	0.483116
26~36	0.298585	0.239206	0.216925	0.285624	0.472481	0.967027	0.726085
26~37	0.322453	0.30629	0.2771	0.340355	0.872834	0.995741	0.161204
26~38	0.30602	0.201309	0.284065	0.242818	0.23249	0.924237	1.214429
26~39	0.362806	0.284184	0.245465	0.303043	0.383279	0.964667	0.882655
26~40	0.312482	0.299104	0.222474	0.307088	0.877756	0.995741	0.154912
26~41	0.303474	0.302431	0.301823	0.289866	0.991412	0.998253	0.010838
26~42	0.308639	0.319967	0.262905	0.270447	0.896636	0.995741	-0.13083
26~43	0.303866	0.257428	0.273676	0.292252	0.616093	0.967027	0.505782
26~44	0.351327	0.33293	0.283055	0.267772	0.838561	0.995414	0.205213
26~45	0.334582	0.286684	0.266136	0.260485	0.579262	0.967027	0.559535
26~46	0.363455	0.317038	0.284284	0.289168	0.621165	0.967027	0.498498
26~47	0.448857	0.462545	0.272064	0.255556	0.874286	0.995741	-0.15935
26~48	0.310669	0.260881	0.183136	0.319808	0.566565	0.967027	0.580388
26~49	0.367546	0.266807	0.247626	0.301708	0.26612	0.924237	1.12959
26~50	0.332947	0.33065	0.222659	0.263945	0.97695	0.998253	0.029094
26~51	0.275286	0.33124	0.264912	0.270531	0.52389	0.967027	-0.64364
26~52	0.326889	0.26501	0.243066	0.260224	0.453473	0.964667	0.757851
26~53	0.263538	0.266059	0.292615	0.298695	0.9792	0.998253	-0.02625
26~54	0.284715	0.264768	0.239807	0.266898	0.809611	0.993329	0.242706
26~55	0.298554	0.296855	0.217147	0.251166	0.982282	0.998253	0.022363
27~28	0.426386	0.412724	0.439611	0.318144	0.914082	0.995741	0.108652
27~29	0.360459	0.423885	0.473895	0.305151	0.631118	0.967027	-0.48428
27~30	0.317534	0.313168	0.456135	0.257007	0.970882	0.998253	0.036803
27~31	0.328318	0.300522	0.371975	0.386744	0.822694	0.994197	0.225722
27~32	0.300846	0.304272	0.381851	0.250916	0.973889	0.998253	-0.03298
27~33	0.397937	0.262886	0.322652	0.274391	0.175554	0.924237	1.381781
27~34	0.373836	0.287922	0.350515	0.35747	0.459683	0.964948	0.747389
27~35	0.388527	0.306855	0.294883	0.310514	0.411241	0.964667	0.831378
27~36	0.37841	0.262521	0.378736	0.430411	0.383094	0.964667	0.883001
27~37	0.370691	0.377461	0.399694	0.249077	0.949909	0.998253	-0.06331
27~38	0.62271	0.43813	0.302655	0.426496	0.129743	0.924237	1.550609
27~39	0.405178	0.393937	0.371978	0.284462	0.917959	0.995741	0.10373
27~40	0.42019	0.28573	0.314163	0.40745	0.259481	0.924237	1.145694
27~41	0.384455	0.333556	0.391272	0.267247	0.639879	0.971576	0.472129
27~42	0.366145	0.226065	0.335839	0.328365	0.202755	0.924237	1.297375
27~43	0.319187	0.263285	0.470833	0.295168	0.667833	0.975425	0.432673
27~44	0.462256	0.344014	0.311171	0.352675	0.27944	0.924237	1.098126

27~45	0.415917	0.263883	0.346377	0.350091	0.187305	0.924237	1.344164
27~46	0.345606	0.226124	0.310067	0.25315	0.204543	0.924237	1.292137
27~47	0.357405	0.280817	0.323449	0.351941	0.488995	0.967027	0.699078
27~48	0.396517	0.290215	0.296234	0.274677	0.260582	0.924237	1.143002
27~49	0.40245	0.278413	0.316069	0.316981	0.235589	0.924237	1.20625
27~50	0.287466	0.212687	0.354928	0.405938	0.548381	0.967027	0.605902
27~51	0.327525	0.353821	0.354043	0.305147	0.808702	0.993329	-0.24389
27~52	0.411014	0.220329	0.43395	0.420414	0.178383	0.924237	1.372552
27~53	0.413215	0.304849	0.450796	0.435563	0.457067	0.964667	0.751787
27~54	0.256727	0.251442	0.415132	0.266193	0.962664	0.998253	0.04717
27~55	0.340839	0.34042	0.31459	0.266552	0.996506	0.998523	0.00441
28~29	0.359385	0.338283	0.435767	0.278306	0.861588	0.995741	0.175606
28~30	0.383817	0.228983	0.319247	0.255628	0.11013	0.924237	1.638019
28~31	0.237962	0.164372	0.317751	0.389469	0.525671	0.967027	0.640863
28~32	0.256039	0.269913	0.304021	0.260219	0.881389	0.995741	-0.15027
28~33	0.30381	0.241362	0.277702	0.266026	0.484726	0.967027	0.706009
28~34	0.433146	0.250822	0.31174	0.369797	0.107924	0.924237	1.648634
28~35	0.349347	0.278939	0.265341	0.284978	0.435484	0.964667	0.788642
28~36	0.298042	0.161843	0.289821	0.372625	0.214159	0.924237	1.264548
28~37	0.383626	0.254998	0.337914	0.291271	0.219391	0.924237	1.249927
28~38	0.432729	0.187019	0.287985	0.358977	0.025054	0.924237	2.338123
28~39	0.309123	0.216123	0.42446	0.272292	0.432623	0.964667	0.793609
28~40	0.361331	0.174695	0.203339	0.367803	0.057499	0.924237	1.962252
28~41	0.371972	0.225726	0.279797	0.299407	0.12837	0.924237	1.556365
28~42	0.287172	0.163548	0.29073	0.312556	0.214651	0.924237	1.263162
28~43	0.413515	0.188312	0.316295	0.281238	0.026819	0.924237	2.3086
28~44	0.348584	0.349135	0.357259	0.302222	0.995956	0.998523	-0.0051
28~45	0.342487	0.199057	0.288963	0.328039	0.160432	0.924237	1.433188
28~46	0.328888	0.215864	0.347964	0.274045	0.277093	0.924237	1.103591
28~47	0.381725	0.331213	0.35123	0.337619	0.654824	0.972006	0.450819
28~48	0.320363	0.194449	0.249441	0.229556	0.115457	0.924237	1.613095
28~49	0.361318	0.186088	0.282214	0.263164	0.056229	0.924237	1.972858
28~50	0.213899	0.163912	0.419003	0.380276	0.703598	0.9857	0.38351
28~51	0.328123	0.301807	0.316443	0.285879	0.790341	0.993329	0.267853
28~52	0.296049	0.142855	0.413521	0.343577	0.225179	0.924237	1.234053
28~53	0.337388	0.138931	0.357827	0.367289	0.100469	0.924237	1.68588
28~54	0.286262	0.185517	0.353123	0.216095	0.291678	0.927465	1.07218
28~55	0.293268	0.273196	0.256468	0.276045	0.817586	0.993329	0.232345
29~30	0.318509	0.308058	0.45642	0.259606	0.932392	0.997713	0.085431
29~31	0.368887	0.389856	0.302382	0.184955	0.800708	0.993329	-0.2543
29~32	0.34534	0.331336	0.286009	0.231976	0.87019	0.995741	0.164587
29~33	0.342723	0.285175	0.331366	0.288902	0.573788	0.967027	0.567662
29~34	0.476568	0.388478	0.25851	0.40231	0.422551	0.964667	0.811254
29~35	0.437547	0.488732	0.307652	0.249117	0.579221	0.967027	-0.55959

29~36	0.33421	0.364669	0.385996	0.249518	0.772404	0.986311	-0.29163
29~37	0.40788	0.477813	0.439412	0.251143	0.556786	0.967027	-0.59315
29~38	0.36854	0.401104	0.374487	0.265911	0.761494	0.986311	-0.30583
29~39	0.641546	0.511467	0.309672	0.352682	0.233879	0.924237	1.210754
29~40	0.376722	0.377631	0.296209	0.260935	0.992079	0.998253	-0.01
29~41	0.500179	0.412216	0.3223	0.265632	0.36787	0.964667	0.911935
29~42	0.376169	0.352381	0.267742	0.226567	0.770541	0.986311	0.293875
29~43	0.371482	0.310479	0.433468	0.267037	0.609584	0.967027	0.515168
29~44	0.390616	0.393506	0.33748	0.266724	0.976974	0.998253	-0.02906
29~45	0.402648	0.33766	0.310048	0.291726	0.51132	0.967027	0.663369
29~46	0.373031	0.319762	0.256724	0.245101	0.518268	0.967027	0.652431
29~47	0.337604	0.406367	0.258483	0.250899	0.411883	0.964667	-0.83023
29~48	0.39635	0.291274	0.269984	0.291687	0.256412	0.924237	1.153239
29~49	0.436519	0.379878	0.252197	0.264058	0.503307	0.967027	0.676085
29~50	0.414144	0.400653	0.283369	0.364257	0.898762	0.995741	0.128126
29~51	0.298646	0.358558	0.417681	0.338002	0.632318	0.967027	-0.48258
29~52	0.374052	0.309326	0.297287	0.289039	0.501502	0.967027	0.678965
29~53	0.303912	0.316579	0.354183	0.289195	0.90522	0.995741	-0.11991
29~54	0.285146	0.370276	0.391951	0.221262	0.410352	0.964667	-0.83472
29~55	0.324615	0.322402	0.314052	0.232055	0.980614	0.998253	0.024468
30~31	0.364341	0.442471	0.501679	0.383235	0.596067	0.967027	-0.53481
30~32	0.526642	0.610715	0.52598	0.316931	0.559969	0.967027	-0.58835
30~33	0.436697	0.34183	0.402771	0.311267	0.425759	0.964667	0.805606
30~34	0.381058	0.360475	0.317735	0.26117	0.829691	0.9943	0.216667
30~35	0.425761	0.376195	0.314115	0.24166	0.592147	0.967027	0.54055
30~36	0.365608	0.345324	0.396128	0.310391	0.862614	0.995741	0.17429
30~37	0.419821	0.328027	0.374074	0.258405	0.38126	0.964667	0.887145
30~38	0.370908	0.272087	0.393418	0.327998	0.408875	0.964667	0.835631
30~39	0.28761	0.353191	0.418983	0.227312	0.558876	0.967027	-0.59
30~40	0.464427	0.362819	0.32341	0.329835	0.344463	0.957459	0.957979
30~41	0.430357	0.369707	0.333849	0.252129	0.535066	0.967027	0.626306
30~42	0.482562	0.525816	0.397555	0.295661	0.708374	0.986311	-0.37702
30~43	0.700461	0.612365	0.355516	0.274166	0.401942	0.964667	0.84818
30~44	0.438002	0.420135	0.393652	0.281383	0.872171	0.995741	0.162108
30~45	0.444705	0.382201	0.361743	0.270848	0.548068	0.967027	0.60656
30~46	0.393889	0.397121	0.383748	0.27159	0.976088	0.998253	-0.03019
30~47	0.337854	0.339864	0.329441	0.339914	0.985336	0.998253	-0.01851
30~48	0.498546	0.438165	0.313721	0.22441	0.503756	0.967027	0.675371
30~49	0.446764	0.365373	0.330761	0.26377	0.410722	0.964667	0.832308
30~50	0.39176	0.431413	0.50474	0.378235	0.787525	0.993329	-0.27154
30~51	0.544866	0.529684	0.386115	0.27371	0.890724	0.995741	0.138364
30~52	0.58022	0.400186	0.364881	0.335328	0.123389	0.924237	1.577673
30~53	0.355685	0.391603	0.481341	0.397436	0.804654	0.993329	-0.24916
30~54	0.498359	0.48638	0.465567	0.302349	0.926513	0.997713	0.092881

30~55	0.475944	0.473746	0.370604	0.280301	0.983813	0.998253	0.02043
31~32	0.53697	0.528646	0.38938	0.417477	0.949647	0.998253	0.063592
31~33	0.420965	0.47703	0.45573	0.286959	0.656976	0.972006	-0.44781
31~34	0.435662	0.452727	0.323811	0.261126	0.860083	0.995741	-0.17754
31~35	0.47242	0.422468	0.325239	0.287677	0.620769	0.967027	0.499066
31~36	0.481921	0.425339	0.28079	0.313684	0.560982	0.967027	0.586827
31~37	0.417062	0.430694	0.344274	0.232719	0.886193	0.995741	-0.14422
31~38	0.396462	0.446218	0.353553	0.255663	0.619875	0.967027	-0.50054
31~39	0.41471	0.347969	0.279803	0.26353	0.455413	0.964667	0.754574
31~40	0.493739	0.477935	0.3741	0.33993	0.892786	0.995741	0.135737
31~41	0.44289	0.435463	0.345258	0.306773	0.944759	0.998253	0.069775
31~42	0.435933	0.470979	0.35716	0.277923	0.736397	0.986311	-0.3393
31~43	0.398992	0.46596	0.377439	0.282401	0.543338	0.967027	-0.6136
31~44	0.461824	0.339553	0.29592	0.453934	0.327146	0.957459	0.9934
31~45	0.495521	0.467797	0.288408	0.294616	0.771301	0.986311	0.292873
31~46	0.425732	0.320874	0.308039	0.29518	0.292375	0.927465	1.068574
31~47	0.417939	0.369129	0.26429	0.302282	0.598605	0.967027	0.531109
31~48	0.517659	0.435064	0.446534	0.377184	0.544225	0.967027	0.612242
31~49	0.508	0.44803	0.320164	0.237056	0.519803	0.967027	0.650024
31~50	0.483483	0.505422	0.288551	0.318119	0.824831	0.994197	-0.22296
31~51	0.301569	0.450708	0.499494	0.444679	0.339691	0.957459	-0.96762
31~52	0.483015	0.462543	0.448677	0.3238	0.874043	0.995741	0.159658
31~53	0.44532	0.429194	0.379897	0.327629	0.889944	0.995741	0.139358
31~54	0.435978	0.49146	0.39546	0.266766	0.612465	0.967027	-0.51134
31~55	0.497931	0.379528	0.331777	0.450681	0.35926	0.964667	0.928641
32~33	0.415534	0.416356	0.468625	0.328584	0.995091	0.998453	-0.0062
32~34	0.433033	0.383635	0.329147	0.270162	0.618559	0.967027	0.502238
32~35	0.491603	0.348789	0.310638	0.298185	0.157928	0.924237	1.442063
32~36	0.403596	0.315873	0.360741	0.357872	0.457362	0.964667	0.751289
32~37	0.404718	0.339637	0.323687	0.236836	0.481231	0.967027	0.712005
32~38	0.346119	0.249452	0.383582	0.314109	0.404181	0.964667	0.844113
32~39	0.331372	0.320843	0.322897	0.23318	0.909812	0.995741	0.114076
32~40	0.449242	0.359503	0.385609	0.381121	0.476028	0.967027	0.720239
32~41	0.415826	0.390809	0.365194	0.277599	0.815045	0.993329	0.235644
32~42	0.587649	0.487364	0.279464	0.324822	0.313139	0.937522	1.022989
32~43	0.482801	0.470156	0.488117	0.346234	0.927887	0.997713	0.09114
32~44	0.460917	0.434271	0.353434	0.271629	0.794866	0.993329	0.261979
32~45	0.444801	0.390208	0.389375	0.265035	0.613761	0.967027	0.509453
32~46	0.535398	0.358872	0.284586	0.260862	0.054735	0.924237	1.985596
32~47	0.375681	0.336105	0.310299	0.288188	0.687178	0.984023	0.405955
32~48	0.449301	0.401049	0.457416	0.27439	0.699781	0.98499	0.38871
32~49	0.475777	0.347378	0.350824	0.196055	0.168614	0.924237	1.410275
32~50	0.435748	0.438368	0.462254	0.413572	0.985476	0.998253	-0.01833
32~51	0.50175	0.616644	0.453361	0.243696	0.344902	0.957459	-0.9571

32~52	0.402825	0.359491	0.598167	0.397723	0.796485	0.993329	0.259818
32~53	0.431191	0.340672	0.461879	0.393699	0.522128	0.967027	0.646388
32~54	0.551769	0.555551	0.44611	0.241255	0.974664	0.998253	-0.03198
32~55	0.531962	0.510083	0.396838	0.340332	0.857064	0.995741	0.181409
33~34	0.416395	0.392742	0.30125	0.284255	0.805407	0.993329	0.248178
33~35	0.478038	0.414085	0.304268	0.333246	0.540168	0.967027	0.618456
33~36	0.471127	0.343774	0.304553	0.296943	0.201067	0.924237	1.302353
33~37	0.392284	0.3337	0.348895	0.277818	0.568803	0.967027	0.575171
33~38	0.42531	0.315845	0.338068	0.250598	0.268767	0.924237	1.123248
33~39	0.366128	0.276938	0.305301	0.30703	0.375796	0.964667	0.896777
33~40	0.445263	0.300835	0.317878	0.29747	0.158113	0.924237	1.441406
33~41	0.409574	0.263194	0.344597	0.328653	0.189839	0.924237	1.336292
33~42	0.391935	0.345344	0.33153	0.311157	0.658801	0.972006	0.445256
33~43	0.383278	0.410662	0.389393	0.308873	0.812982	0.993329	-0.23832
33~44	0.461264	0.387326	0.330954	0.339176	0.501099	0.967027	0.679609
33~45	0.537419	0.500312	0.362442	0.353282	0.751643	0.986311	0.318903
33~46	0.353904	0.264615	0.298894	0.305752	0.369103	0.964667	0.909563
33~47	0.35349	0.300852	0.280148	0.311513	0.586722	0.967027	0.548518
33~48	0.476188	0.335874	0.318795	0.367694	0.215772	0.924237	1.260011
33~49	0.501118	0.395617	0.299531	0.273499	0.266219	0.924237	1.129352
33~50	0.252896	0.315126	0.387483	0.327266	0.598264	0.967027	-0.53161
33~51	0.37772	0.339202	0.343314	0.316968	0.722416	0.986311	0.358025
33~52	0.472491	0.292496	0.395443	0.351799	0.148763	0.924237	1.475531
33~53	0.349999	0.261317	0.467918	0.31895	0.504082	0.967027	0.674851
33~54	0.30881	0.291942	0.445422	0.307142	0.893843	0.995741	0.13439
33~55	0.42844	0.297187	0.317084	0.351473	0.234012	0.924237	1.210401
34~35	0.675964	0.518355	0.237232	0.51666	0.247359	0.924237	1.186545
34~36	0.443374	0.351403	0.234276	0.291056	0.288246	0.924237	1.077904
34~37	0.432511	0.387091	0.336999	0.335237	0.679975	0.98051	0.415868
34~38	0.390601	0.360119	0.318369	0.226668	0.738485	0.986311	0.336449
34~39	0.450686	0.41996	0.293614	0.285248	0.74597	0.986311	0.326456
34~40	0.45579	0.378035	0.207919	0.284888	0.339562	0.957459	0.96788
34~41	0.468067	0.431461	0.301657	0.320069	0.718816	0.986311	0.362881
34~42	0.493646	0.480999	0.277887	0.279608	0.8897	0.995741	0.139668
34~43	0.395327	0.372677	0.31703	0.270295	0.815061	0.993329	0.235622
34~44	0.484095	0.489002	0.444145	0.413659	0.972176	0.998253	-0.03512
34~45	0.46941	0.466299	0.312642	0.279636	0.974497	0.998253	0.032191
34~46	0.480661	0.446629	0.405025	0.307506	0.774126	0.987609	0.28915
34~47	0.495164	0.549354	0.404019	0.278473	0.63693	0.9691	-0.47603
34~48	0.545122	0.382428	0.229692	0.338632	0.088851	0.924237	1.748789
34~49	0.573154	0.43157	0.246154	0.361184	0.162902	0.924237	1.424541
34~50	0.435869	0.575521	0.274268	0.208952	0.088606	0.924237	-1.75018
34~51	0.366072	0.446426	0.308521	0.3449	0.453274	0.964667	-0.75819
34~52	0.461296	0.41317	0.253466	0.234808	0.548963	0.967027	0.605017

34~53	0.34213	0.357217	0.368922	0.267105	0.885247	0.995741	-0.14539
34~54	0.355576	0.403463	0.325918	0.245581	0.615302	0.967027	-0.50692
34~55	0.420487	0.304536	0.266002	0.339161	0.246216	0.924237	1.178786
35~36	0.469718	0.304736	0.21021	0.325018	0.068666	0.924237	1.876825
35~37	0.467327	0.441223	0.309446	0.335789	0.804468	0.993329	0.249402
35~38	0.396397	0.264294	0.301271	0.320471	0.198636	0.924237	1.309576
35~39	0.450318	0.398385	0.238677	0.357168	0.60629	0.967027	0.521011
35~40	0.474053	0.298088	0.245879	0.329875	0.074108	0.924237	1.847837
35~41	0.483734	0.431016	0.293	0.350013	0.616497	0.967027	0.505201
35~42	0.501757	0.423382	0.25564	0.251034	0.347593	0.957459	0.951705
35~43	0.433586	0.390526	0.327277	0.242565	0.650821	0.972006	0.456432
35~44	0.530834	0.515899	0.279522	0.261262	0.866282	0.995741	0.16959
35~45	0.51634	0.432704	0.315361	0.395262	0.473471	0.967027	0.724452
35~46	0.480404	0.38281	0.242313	0.275495	0.252885	0.924237	1.16199
35~47	0.440838	0.429801	0.267802	0.29889	0.905085	0.995741	0.120083
35~48	0.581992	0.490944	0.222557	0.297239	0.289196	0.924237	1.075751
35~49	0.679034	0.706074	0.21991	0.221466	0.708243	0.986311	-0.3772
35~50	0.46303	0.377914	0.239695	0.421826	0.443554	0.964667	0.774737
35~51	0.415691	0.38385	0.290564	0.335381	0.755647	0.986311	0.313584
35~52	0.479572	0.265352	0.27997	0.319296	0.034016	0.924237	2.203838
35~53	0.358315	0.265637	0.33426	0.330636	0.396689	0.964667	0.85778
35~54	0.412568	0.344063	0.319723	0.307641	0.506285	0.967027	0.671347
35~55	0.447145	0.331025	0.276868	0.218316	0.162956	0.924237	1.424352
36~37	0.462686	0.460262	0.366283	0.305209	0.982554	0.998253	0.02202
36~38	0.455659	0.526207	0.394139	0.343935	0.562298	0.967027	-0.58485
36~39	0.395534	0.442344	0.330777	0.245163	0.626447	0.967027	-0.49094
36~40	0.498774	0.545907	0.350479	0.33387	0.674613	0.978321	-0.42327
36~41	0.463839	0.422666	0.325283	0.30378	0.690128	0.984023	0.401908
36~42	0.397589	0.318315	0.321912	0.229647	0.384869	0.964667	0.880214
36~43	0.382306	0.365526	0.386379	0.208512	0.867079	0.995741	0.16882
36~44	0.412041	0.242998	0.307833	0.425952	0.166397	0.924237	1.412476
36~45	0.471097	0.372704	0.302195	0.285931	0.310857	0.936999	1.027893
36~46	0.336147	0.175199	0.289608	0.269287	0.085535	0.924237	1.768004
36~47	0.35935	0.244221	0.253926	0.303186	0.211035	0.924237	1.273408
36~48	0.475933	0.372333	0.265071	0.255739	0.229232	0.924237	1.223116
36~49	0.474052	0.313083	0.285468	0.301095	0.09943	0.924237	1.69125
36~50	0.293336	0.380904	0.394491	0.30725	0.453874	0.964667	-0.75717
36~51	0.330029	0.248437	0.363012	0.420493	0.524978	0.967027	0.641941
36~52	0.478294	0.46813	0.33947	0.300096	0.923016	0.997713	0.097314
36~53	0.375821	0.438206	0.4112	0.305948	0.596816	0.967027	-0.53388
36~54	0.336352	0.315107	0.376756	0.249244	0.837387	0.99525	0.206855
36~55	0.456884	0.382858	0.319127	0.413426	0.538277	0.967027	0.621361
37~38	0.441753	0.461218	0.395277	0.222834	0.851094	0.995741	-0.18932
37~39	0.442119	0.61192	0.482514	0.253434	0.19018	0.924237	-1.33524

37~40	0.444677	0.511091	0.3439	0.236564	0.489213	0.967027	-0.6992
37~41	0.532387	0.645007	0.405582	0.262515	0.312484	0.937522	-1.02579
37~42	0.390239	0.317074	0.342324	0.263677	0.468987	0.967027	0.731868
37~43	0.465591	0.344947	0.358018	0.264899	0.249817	0.924237	1.169676
37~44	0.415161	0.337238	0.323142	0.312547	0.455878	0.964667	0.753789
37~45	0.407188	0.368785	0.371314	0.31468	0.734415	0.986311	0.341899
37~46	0.354875	0.240459	0.318375	0.31381	0.272816	0.924237	1.113635
37~47	0.350377	0.262243	0.272343	0.314337	0.360572	0.964667	0.926079
37~48	0.414424	0.444255	0.392754	0.283131	0.788429	0.993329	-0.27045
37~49	0.475766	0.414015	0.358433	0.306171	0.573728	0.967027	0.567751
37~50	0.292811	0.316748	0.466895	0.310439	0.855149	0.995741	-0.18387
37~51	0.386504	0.348814	0.380114	0.257329	0.720401	0.986311	0.360949
37~52	0.354248	0.330439	0.422787	0.246997	0.831597	0.9943	0.214447
37~53	0.303482	0.35752	0.4295	0.219257	0.62401	0.967027	-0.49548
37~54	0.366979	0.328397	0.381936	0.243704	0.710195	0.986311	0.374855
37~55	0.410085	0.359864	0.317899	0.225694	0.581887	0.967027	0.55565
38~39	0.442545	0.430985	0.337907	0.301534	0.912422	0.995741	0.11076
38~40	0.475962	0.436516	0.291033	0.348512	0.706143	0.986311	0.380047
38~41	0.42035	0.345944	0.352923	0.295683	0.488394	0.967027	0.700052
38~42	0.394109	0.272731	0.347855	0.220523	0.203607	0.924237	1.297453
38~43	0.397327	0.294591	0.397217	0.259754	0.347844	0.957459	0.952343
38~44	0.428605	0.181304	0.35313	0.430628	0.059799	0.924237	1.943555
38~45	0.425877	0.307374	0.383926	0.339142	0.322309	0.94778	1.003517
38~46	0.34632	0.217236	0.339639	0.229535	0.175399	0.924237	1.384342
38~47	0.346735	0.278633	0.323549	0.272012	0.489674	0.967027	0.69798
38~48	0.399158	0.282616	0.317128	0.362648	0.297582	0.928415	1.056936
38~49	0.415296	0.314638	0.330129	0.297968	0.332434	0.957459	0.982453
38~50	0.33838	0.360784	0.38809	0.312954	0.846903	0.995741	-0.19447
38~51	0.365428	0.21685	0.341905	0.419029	0.236993	0.924237	1.202571
38~52	0.404549	0.454092	0.486008	0.29307	0.709745	0.986311	-0.37516
38~53	0.48223	0.3967	0.395439	0.31788	0.47045	0.967027	0.729443
38~54	0.325788	0.277686	0.392509	0.257928	0.65526	0.972006	0.450539
38~55	0.415269	0.264062	0.343644	0.398434	0.217183	0.924237	1.256065
39~40	0.400876	0.415351	0.290616	0.232435	0.867296	0.995741	-0.16829
39~41	0.531029	0.469232	0.286916	0.270921	0.500492	0.967027	0.68058
39~42	0.345362	0.38176	0.357666	0.216586	0.710503	0.986311	-0.37413
39~43	0.264861	0.343989	0.433796	0.229299	0.481698	0.967027	-0.7126
39~44	0.413231	0.364354	0.284426	0.268237	0.590267	0.967027	0.543308
39~45	0.436138	0.373114	0.290682	0.268495	0.493533	0.967027	0.691749
39~46	0.407107	0.308025	0.214177	0.272179	0.218106	0.924237	1.253495
39~47	0.355308	0.327756	0.227677	0.277688	0.738967	0.986311	0.335805
39~48	0.451655	0.422116	0.260674	0.245841	0.72224	0.986311	0.358261
39~49	0.458877	0.389071	0.28323	0.340794	0.495104	0.967027	0.689219
39~50	0.416784	0.427306	0.324621	0.266333	0.914208	0.995741	-0.10849

39~51	0.298568	0.363273	0.419762	0.263917	0.578055	0.967027	-0.56132
39~52	0.420376	0.369318	0.288136	0.216466	0.544404	0.967027	0.611969
39~53	0.30413	0.324943	0.379393	0.253902	0.842328	0.995741	-0.20047
39~54	0.242532	0.360009	0.428107	0.257336	0.307772	0.936999	-1.03663
39~55	0.345102	0.312801	0.290457	0.217476	0.70288	0.9857	0.384486
40~41	0.494457	0.52676	0.298789	0.287082	0.736601	0.986311	-0.33897
40~42	0.474289	0.363464	0.300499	0.252303	0.229055	0.924237	1.223592
40~43	0.430154	0.379195	0.358589	0.240045	0.606995	0.967027	0.519276
40~44	0.442684	0.296999	0.30518	0.410841	0.219674	0.924237	1.249143
40~45	0.46587	0.379027	0.311405	0.284993	0.377627	0.964667	0.893305
40~46	0.417429	0.200894	0.26707	0.314106	0.027583	0.924237	2.296358
40~47	0.395078	0.297063	0.236248	0.29906	0.267315	0.924237	1.126721
40~48	0.535192	0.399707	0.245974	0.315464	0.146418	0.924237	1.484358
40~49	0.506963	0.284623	0.264108	0.328006	0.026613	0.924237	2.311946
40~50	0.397355	0.409556	0.347557	0.325652	0.911992	0.995741	-0.11131
40~51	0.469988	0.325382	0.292142	0.434324	0.23215	0.924237	1.215331
40~52	0.556184	0.461395	0.336127	0.35435	0.403129	0.964667	0.846022
40~53	0.405526	0.476468	0.420032	0.318682	0.559054	0.967027	-0.58988
40~54	0.403937	0.358669	0.398399	0.267647	0.681013	0.98051	0.414696
40~55	0.536031	0.425811	0.362286	0.445428	0.406242	0.964667	0.840382
41~42	0.425128	0.375373	0.325759	0.253533	0.605477	0.967027	0.521117
41~43	0.406861	0.359175	0.357169	0.282128	0.653169	0.972006	0.453138
41~44	0.430552	0.355665	0.351864	0.308592	0.492141	0.967027	0.693993
41~45	0.50298	0.367143	0.296801	0.318921	0.182314	0.924237	1.359911
41~46	0.368495	0.255937	0.321874	0.321633	0.288766	0.924237	1.076724
41~47	0.355552	0.297591	0.293107	0.3108	0.557867	0.967027	0.591522
41~48	0.513009	0.47759	0.313507	0.297829	0.723897	0.986311	0.356029
41~49	0.518781	0.409092	0.30349	0.32359	0.288135	0.924237	1.078157
41~50	0.396449	0.381949	0.301928	0.269427	0.877311	0.995741	0.155481
41~51	0.408994	0.411177	0.334928	0.260559	0.982376	0.998253	-0.02224
41~52	0.463259	0.370946	0.320554	0.23596	0.315951	0.942143	1.017513
41~53	0.373626	0.333154	0.396856	0.302181	0.724211	0.986311	0.355678
41~54	0.370404	0.369526	0.35017	0.257242	0.993086	0.998453	0.008725
41~55	0.436301	0.406159	0.315974	0.222857	0.738664	0.986311	0.33621
42~43	0.5441	0.554626	0.482419	0.332657	0.938714	0.998253	-0.07743
42~44	0.510319	0.421965	0.331976	0.394275	0.458292	0.964667	0.749725
42~45	0.494035	0.432801	0.299535	0.292986	0.528961	0.967027	0.63575
42~46	0.669589	0.534879	0.29789	0.295696	0.171044	0.924237	1.39674
42~47	0.464694	0.470326	0.290991	0.282167	0.952145	0.998253	-0.06043
42~48	0.52175	0.390751	0.305252	0.251564	0.160209	0.924237	1.433976
42~49	0.505769	0.386502	0.283654	0.226938	0.164066	0.924237	1.420498
42~50	0.500273	0.531001	0.398956	0.316305	0.795527	0.993329	-0.26107
42~51	0.568412	0.449651	0.320715	0.424831	0.334285	0.957459	0.978647
42~52	0.474289	0.380781	0.441717	0.282554	0.447876	0.964667	0.767351

42~53	0.437062	0.381859	0.408282	0.297669	0.640073	0.971576	0.471579
42~54	0.520601	0.472441	0.414816	0.279625	0.680788	0.98051	0.414748
42~55	0.445459	0.357868	0.359606	0.332781	0.442524	0.964667	0.776503
43~44	0.469619	0.404992	0.380743	0.323317	0.57849	0.967027	0.560678
43~45	0.416336	0.46892	0.404661	0.284714	0.643626	0.972006	-0.46678
43~46	0.402164	0.389398	0.482337	0.340434	0.926213	0.997713	0.093261
43~47	0.367289	0.376179	0.328037	0.345744	0.935641	0.997713	-0.08132
43~48	0.432283	0.400306	0.357667	0.22884	0.747769	0.986311	0.324059
43~49	0.429894	0.338047	0.347559	0.224127	0.335532	0.957459	0.977392
43~50	0.484043	0.431097	0.480113	0.317829	0.694448	0.984023	0.395992
43~51	0.472474	0.421314	0.537184	0.338482	0.725047	0.986311	0.354787
43~52	0.541221	0.419284	0.435032	0.267298	0.300629	0.930327	1.052116
43~53	0.51816	0.343814	0.37764	0.293888	0.123952	0.924237	1.575229
43~54	0.626579	0.409501	0.321447	0.28464	0.034821	0.924237	2.193386
43~55	0.521229	0.372232	0.300992	0.315378	0.145039	0.924237	1.489599
44~45	0.582697	0.39881	0.340688	0.426482	0.148778	0.924237	1.475476
44~46	0.539757	0.405044	0.283216	0.313454	0.172471	0.924237	1.391972
44~47	0.607846	0.518513	0.273201	0.339328	0.37518	0.964667	0.897948
44~48	0.483348	0.366947	0.30035	0.292029	0.234714	0.924237	1.208551
44~49	0.505419	0.382861	0.324485	0.294468	0.232566	0.924237	1.214228
44~50	0.4112	0.360959	0.283873	0.428095	0.669452	0.975425	0.430426
44~51	0.364813	0.483253	0.329408	0.291726	0.250584	0.924237	-1.16775
44~52	0.465265	0.283798	0.366183	0.423179	0.165031	0.924237	1.417168
44~53	0.412721	0.256792	0.361285	0.44758	0.24301	0.924237	1.186979
44~54	0.38945	0.36728	0.346142	0.321946	0.839709	0.995741	0.203733
44~55	0.426635	0.401858	0.344071	0.287646	0.812222	0.993329	0.23931
45~46	0.404304	0.293444	0.296029	0.333949	0.28516	0.924237	1.084941
45~47	0.445658	0.332979	0.267399	0.334378	0.256659	0.924237	1.152628
45~48	0.493607	0.416882	0.292902	0.327721	0.450902	0.964667	0.762206
45~49	0.546507	0.430767	0.3256	0.284273	0.253189	0.924237	1.161234
45~50	0.393079	0.433949	0.281571	0.255988	0.643838	0.972006	-0.46626
45~51	0.39222	0.337116	0.333458	0.354703	0.624629	0.967027	0.493539
45~52	0.513953	0.32148	0.33947	0.312533	0.078409	0.924237	1.811528
45~53	0.40172	0.32606	0.386981	0.285766	0.494796	0.967027	0.68997
45~54	0.393422	0.37731	0.362455	0.25176	0.873512	0.995741	0.160406
45~55	0.452692	0.269949	0.32458	0.363925	0.110468	0.924237	1.636409
46~47	0.585561	0.657156	0.272678	0.217281	0.380252	0.964667	-0.88835
46~48	0.483321	0.260126	0.271111	0.311544	0.023731	0.924237	2.361517
46~49	0.458505	0.2759	0.286115	0.281124	0.055312	0.924237	1.980646
46~50	0.460019	0.440756	0.41497	0.335383	0.876711	0.995741	0.156247
46~51	0.485312	0.396282	0.371334	0.309273	0.430149	0.964667	0.79792
46~52	0.418327	0.252237	0.44258	0.309643	0.193221	0.924237	1.32591
46~53	0.362956	0.218748	0.451083	0.292502	0.255869	0.924237	1.154581
46~54	0.40865	0.35188	0.459487	0.287064	0.654948	0.972006	0.450644

46~55	0.377573	0.303275	0.358322	0.275807	0.482158	0.967027	0.710196
47~48	0.40709	0.291575	0.27907	0.361795	0.275081	0.924237	1.108303
47~49	0.423427	0.312323	0.283948	0.327141	0.269784	0.924237	1.120823
47~50	0.34608	0.453978	0.275751	0.277176	0.237432	0.924237	-1.20142
47~51	0.313277	0.366782	0.328319	0.33546	0.622579	0.967027	-0.49647
47~52	0.393328	0.328204	0.283669	0.306056	0.500388	0.967027	0.680745
47~53	0.300265	0.287903	0.330716	0.305363	0.905713	0.995741	0.119285
47~54	0.342552	0.332858	0.316421	0.287693	0.922151	0.997713	0.098412
47~55	0.37686	0.247488	0.258826	0.30687	0.167302	0.924237	1.409384
48~49	0.627325	0.596161	0.274876	0.328924	0.752293	0.986311	0.318039
48~50	0.460928	0.345996	0.350934	0.293916	0.283995	0.924237	1.087612
48~51	0.530522	0.37889	0.290697	0.247679	0.093889	0.924237	1.720709
48~52	0.595713	0.327714	0.27573	0.322664	0.009022	0.924237	2.760532
48~53	0.392949	0.334465	0.456388	0.291693	0.645005	0.972006	0.464615
48~54	0.431924	0.400655	0.454955	0.270679	0.801125	0.993329	0.253759
48~55	0.446724	0.347633	0.351074	0.263583	0.336079	0.957459	0.974975
49~50	0.455444	0.377083	0.276415	0.281692	0.392913	0.964667	0.86473
49~51	0.433545	0.336125	0.319044	0.247562	0.303935	0.933062	1.042928
49~52	0.519891	0.288317	0.294367	0.295262	0.020799	0.924237	2.417882
49~53	0.413256	0.287255	0.36075	0.313232	0.260358	0.924237	1.143549
49~54	0.418356	0.355404	0.374608	0.269646	0.559861	0.967027	0.588515
49~55	0.483857	0.259832	0.284597	0.269292	0.017737	0.924237	2.485036
50~51	0.504497	0.593429	0.649196	0.464198	0.633604	0.967027	-0.48075
50~52	0.570287	0.516911	0.398325	0.269358	0.635354	0.967692	0.478265
50~53	0.535973	0.422958	0.366632	0.41164	0.376579	0.964667	0.895292
50~54	0.639307	0.537393	0.442239	0.319834	0.425623	0.964667	0.805845
50~55	0.463929	0.367672	0.334158	0.449996	0.455981	0.964667	0.753615
51~52	0.533787	0.350345	0.504193	0.434367	0.23992	0.924237	1.194948
51~53	0.341918	0.365134	0.57662	0.473972	0.893629	0.995741	-0.13466
51~54	0.580148	0.607414	0.615705	0.351463	0.869795	0.995741	-0.16509
51~55	0.445616	0.512825	0.399285	0.282557	0.557136	0.967027	-0.59263
52~53	0.508148	0.484894	0.601923	0.355001	0.887135	0.995741	0.14294
52~54	0.501021	0.377989	0.525801	0.32414	0.397445	0.964667	0.856394
52~55	0.564755	0.464144	0.381216	0.43817	0.453997	0.964667	0.756964
53~54	0.532561	0.418217	0.397974	0.314619	0.336136	0.957459	0.974858
53~55	0.450174	0.366313	0.352797	0.468145	0.534267	0.967027	0.627538
54~55	0.540139	0.404132	0.304456	0.289146	0.16772	0.924237	1.40796
